# Mining 3D genome structure populations identifies major factors governing the stability of regulatory communities

**DOI:** 10.1038/ncomms11549

**Published:** 2016-05-31

**Authors:** Chao Dai, Wenyuan Li, Harianto Tjong, Shengli Hao, Yonggang Zhou, Qingjiao Li, Lin Chen, Bing Zhu, Frank Alber, Xianghong Jasmine Zhou

**Affiliations:** 1 Molecular and Computational Biology, Department of Biological Sciences, University of Southern California, 1050 Childs Way, Los Angeles, California 90089, USA; 2 National Laboratory of Biomacromolecules, Institute of Biophysics, Chinese Academy of Sciences, Beijing 100101, China

## Abstract

Three-dimensional (3D) genome structures vary from cell to cell even in an isogenic sample. Unlike protein structures, genome structures are highly plastic, posing a significant challenge for structure-function mapping. Here we report an approach to comprehensively identify 3D chromatin clusters that each occurs frequently across a population of genome structures, either deconvoluted from ensemble-averaged Hi-C data or from a collection of single-cell Hi-C data. Applying our method to a population of genome structures (at the macrodomain resolution) of lymphoblastoid cells, we identify an atlas of stable inter-chromosomal chromatin clusters. A large number of these clusters are enriched in binding of specific regulatory factors and are therefore defined as ‘Regulatory Communities.' We reveal two major factors, centromere clustering and transcription factor binding, which significantly stabilize such communities. Finally, we show that the regulatory communities differ substantially from cell to cell, indicating that expression variability could be impacted by genome structures.

## Abstract (web summary)

3D genome structures are plastic and vary from cell to cell even in an isogenic sample. Here, the authors present an approach to identify frequent 3D chromatin clusters across a population of genome structures, either deconvoluted from ensemble-averaged Hi-C data or from a collection of single-cell Hi-C data.

## Graphical abstract

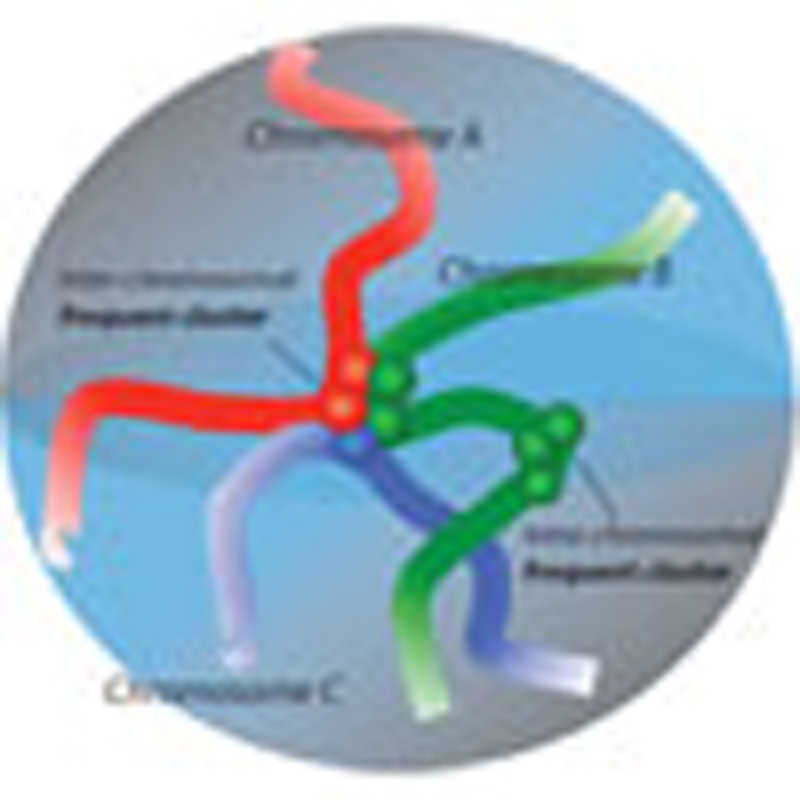

Genome-wide proximity ligation assays, such as Hi-C^1^ and its variants^2^,^3^,^4^, as well as ChIA-PET^5^ have significantly expanded our understanding of spatial genome organization. Yet our knowledge of how genome structures are linked to functions is still limited. For example, recent studies have uncovered functional roles for local chromatin looping interactions^6^,^7^,^8^,^9^. However, few studies have tried to decipher the functional implications of inter-chromosomal associations, which are known to play important roles in gene regulation. We can cite three examples that demonstrate the importance of inter-chromosomal interactions in gene regulation: inter-chromosomal contacts are required for co-transcription of the multi-gene complex *SAMD4A*, *TNFAIP2* and *SLC6A5* (ref. 10); the *IFN-γ* gene on chromosome 10 is trans-activated by regulatory regions of the *T*_*H*_*2* cytokine locus on chromosome 11 (ref. 11); and *INF-beta* genes are trans-activated from regulatory regions in different chromosomes^12^.

A challenge arises when using Hi-C data to infer the spatial genome organization linked to inter-chromosomal functional interactions. The chromatin contacts uncovered by Hi-C describe not a single genome conformation, but the average contact frequency over many different genome conformations in a population of cells^13^,^14^,^15^. The spatial genome organization can vary dramatically from cell to cell, even within an isogenic sample^16^. This variability is especially strong for inter-chromosomal contacts. Therefore, a key challenge for interpreting ensemble-averaged Hi-C data is to deconvolute the observed chromatin contacts into an ensemble of subsets of interactions, where each subset are compatible to co-occur in a single cell.

To address this challenge, we have recently developed a modelling approach that constructs a population of three-dimensional (3D) genome structures derived from and fully consistent with the Hi-C data^3^,^17^. By embedding the genome structural model in 3D space and applying additional spatial constraints (for example, all chromosomes must lie within the nuclear volume, and no two chromosome fragments can overlap), it is possible to deconvolute the ensemble-based Hi-C data into a set of plausible structural states. In particular, our approach facilitates the inference of cooperative long-range interactions, because the presence of some chromatin contacts may induce structural features that may make some additional contacts in the same structure more probable and others less likely. In other words, the spatial constraints effectively restrict the conformational freedom of the chromosomes, allowing us to approximate the unobserved true structure population. We showed previously that the structure population determined by this method can reproduce remarkably well independent experimental data and many known structural features of the genome organization that were not directly evident in the Hi-C data^3^,^17^,^18^,^19^.

The analysis of a genome structure population (either from population-based modelling or in future from a large collection of single-cell Hi-C data) demands a new generation of structural analysis tools. Unlike protein structures, genome structures are highly plastic^15^,^20^,^21^. Therefore, traditional measures of structural similarities such as structure alignments based on the root-mean-square deviations of chromatin positions are not suitable. We need to distinguish functional chromatin interactions from noise in the Hi-C data, and also from random chromatin collisions, which together occupy a large percentage of the measured signals.

This paper develops a novel computational framework to derive an atlas of functional chromatin clusters from the modelled genome structure population. Our rationale is that despite the existence of large cell-to-cell variations between genome structures, any functionally important spatial patterns should generally occur in a reasonable percentage of cells (in this paper, the threshold is 1%). That is, given a population of genome structures, we assume that spatial patterns occurring in multiple structures are more likely to be of functional importance. The most obvious example of functional spatial patterns would be a spatial chromatin cluster that brings together several distant loci. Such clusters have already been found and associated with co-transcription factories^16^,^22^,^23^, early-replication sites^24^,^25^,^26^ and chromatin silencing^27^. Here we show that our approach can comprehensively detect spatial chromatin clusters that frequently occur in a genome structure population.

We apply our pattern discovery method to a population of 10,000 genome structures at the resolution of macrodomains, inferred from the tethered chromosome conformation capture (TCC^3^, a variant of Hi-C) data of human lymphoblastoid cells. This analysis identifies an atlas of spatial clusters (of which a substantial portion is inter-chromosomal) that occur frequently across the structure population. Our 3D fluorescence *in situ* hybridization (FISH) experiments validate the pronounced co-localizations of domains in two identified inter-chromosomal clusters. Strikingly, a significant portion of those inter-chromosomal frequent clusters are enriched in binding of specific regulatory factors, which are defined as ‘Regulatory Communities', including transcription communities, transfer RNA (tRNA) synthesis communities, polycomb binding communities and many others, depending on the type of binding factors. Interestingly, the regulatory communities carrying the same function (for example, with the same binding factors) can exchange their members, indicating that the structural plasticity of the human genome may outweigh its functional plasticity. It is important to emphasize that the majority of these communities cannot be identified directly from the Hi-C heat map, but are only evident after the structure-based deconvolution of the Hi-C data, since the Hi-C data provides only binary contacts averaged across many cells, and not the higher order chromatin contacts (involving more than two loci) in any single cells. We reveal that two major factors, centromere clustering and transcription factor binding, significantly contribute to the stability of the 3D regulatory communities in lymphoblastoid cells. We further show that these regulatory communities differ dramatically from cell to cell, indicating that expression variability could be impacted by the 3D genome structures.

## Results

### Discover frequent spatial clusters in a 3D genome population

We performed the TCC^3^, a variant of Hi-C, in human lymphoblastoid cells. After data pre-processing and normalization, we performed constrained clustering^28^ of the Hi-C contact map to partition the linear genome into 428 structural domains as described previously^3^. The genomic loci within each domain share similar contact profiles. The median domain size is 5.2 Mb (the min and max domain size are 0.3 Mb and 50 Mb, respectively). We categorized 63 domains as centromeric domains since they overlap sub-centromeric regions (5 Mb up- and downstream to the centromere locations). We classify the remaining domains into transcription active (126), inactive (141) and others (98) based on hierarchal clustering of the ChIP-seq signal of 12 epigenetic/transcriptional marks (Supplementary Fig. 1). Our 3D model of the genome treats these domains as spheres with radii that approximate the domain sizes^3^. We used the Hi-C data to perform our population-based structural modelling^3^ at this domain resolution, and generated 10,000 3D genome structures whose chromatin contacts reproduce the domain contact frequencies obtained from the Hi-C experiment. Our method performs a structure-based deconvoution of the Hi-C map into individual genome structures, which represent the best approximation of the true structure population given the available data. We have previously demonstrated that a population of 10,000 structures is sufficiently large to describe cell-to-cell variations, and that the resulting structure population reproduces remarkably well many known properties of the genome^3^,^17^.

Given the 10,000 genome structures, we developed a graph-based mining approach to identify frequent spatial clusters, defined as a set of chromatin domains (from one or multiple chromosomes) that co-localize in many genome structures. Since the human diploid genome has homologous chromosomes, each genome structure consists of 2*N* (*N*=428) homologous domains. Therefore, each 3D structure can be modelled as a chromatin interaction graph (CIG) with 2*N* nodes, where each node is a domain and each edge represents an interaction between two domains (details in Supplementary Note 1). A unique feature of the CIG is that each node has three labels: L1 is the index of the chromosome where the domain is located; L2 is the index of the domain among all domains in the chromosome; and L3 indicates which copy of the chromosome the domain comes from (Supplementary Fig. 2). Therefore, each node of a CIG is labelled by a triplet L1–L2–L3, in which the L1 and L3 labels can tell us whether two homologous domains reside in the same chromosome copy. For example, nodes 1-1-A and 1-3-B represent two different domains located in different copies of the chromosome 1. After representing each 3D genome structure as a CIG, discovering frequent spatial clusters can be transformed to the problem of discovering frequent dense subgraphs across the CIG*s*. However, the diploid nature of the human genome adds a novel feature to the subgraph identification problem: when a cluster contains two or more domains from the same chromosome, we need to differentiate between domains residing in the same or different chromosome copies. We refer to this feature of CIGs as ‘coupled isomorphism', which is different from the classic ‘graph isomorphism'. To our knowledge, coupled isomorphism has not been seen in any existing graph problem (for a detailed explanation refer to Supplementary Fig. 2). Our goal is to identify subsets of the domains that are densely connected and frequently occur across the given set of graphs, after taking into account the coupled isomorphism. The unique property of the coupled isomorphism, along with the large number and scale of the graphs (tens of thousands of graphs, each with hundreds to thousands of nodes), poses a great challenge.

**Figure 1:**
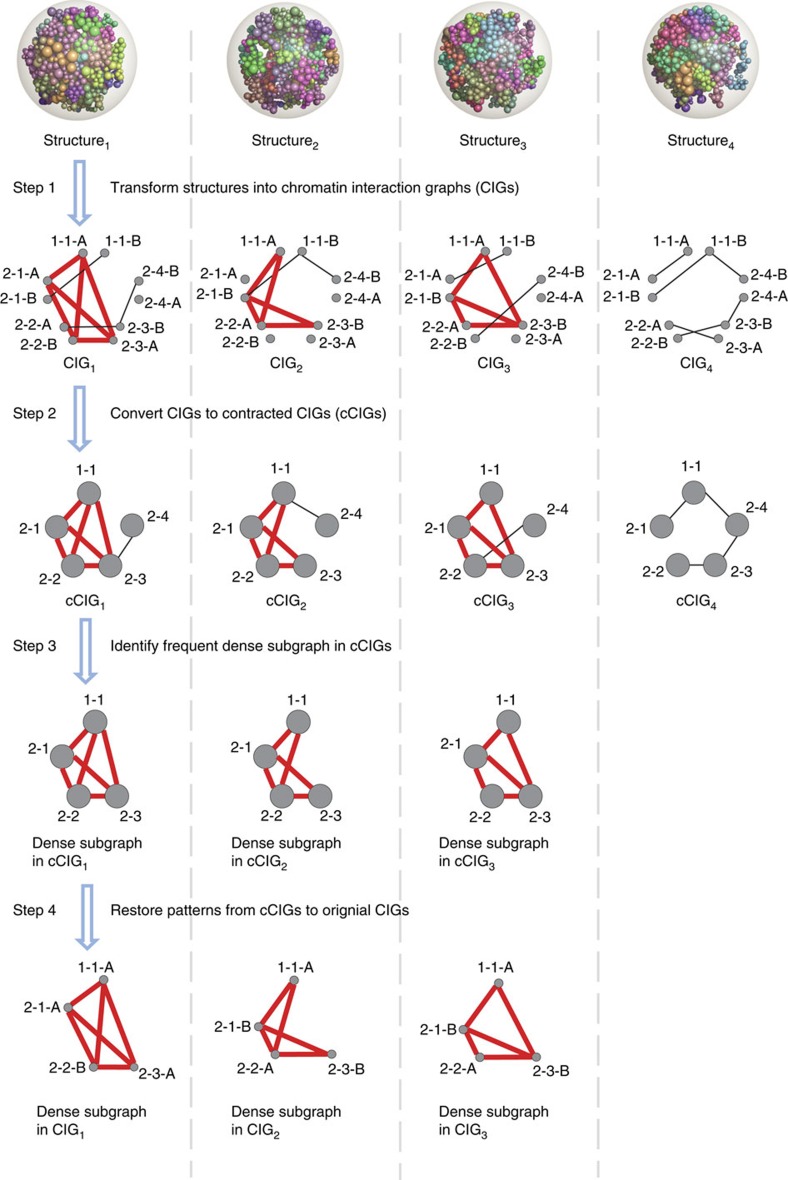
The overall procedure to discover frequent dense clusters. Each 3D genome structure is transformed into a CIG where a node represents a domain and an edge represents a contact between two domains. In this example, 4 CIGs with 10 nodes are built. Each node is labelled by the triplet L1–L2–L3, where L1 indicates the chromosome index, L2 indicates the domain index among all domains in its chromosome and L3 (a letter A or B) indicates which copy of the chromosome the domain comes from. For example, nodes 2-3-A and 2-3-B indicate ‘twin' nodes are from the 3rd domain of chromosome 2, in its two homologous copies. Step 1: Each genome structure is transformed into a CIG. Step 2: in each CIG, we merge any two ‘twin' nodes that represent homologous domains. This step yields a collection of four contracted graphs without isomorphism (termed cCIG, where each node is a merged domain, labelled by L1 and L2). Step 3: We identify the dense subgraphs that frequently occur across many networks using a tensor-based computational method. Step 4: We restore each frequent dense subgraph to its un-contracted form in the original CIG*s*, and after mining on these subgraphs with ‘coupled isomorphism' we identify the final set of frequent dense subgraphs.

We have proposed a four-step computational framework to address this novel graph mining problem. First, we transformed a population of genome structures into a series of CIGs. Second, we applied the node contraction technique to reduce ‘coupled isomorphism' by converting the multi-labelled graphs into graphs without L3 labels. The resulting contracted graphs are guaranteed to preserve all occurrences of frequent patterns in the original graphs^29^. This technique postpones the problem of resolving subgraph isomorphism to a later step when we will only analyse small candidate patterns, effectively reducing the problem complexity. Third, we employed an efficient and scalable algorithm for frequent subgraph discovery on the contracted graphs^30^. This algorithm requires very few *ad hoc* parameters, and is capable of simultaneously analysing a large number of massive graphs. It works by reformulating the subgraph discovery problem as a tensor-based optimization problem. In the last step, after obtaining a collection of frequent dense subgraphs from the contracted graphs, we identify the counterparts of these patterns in the original multi-labelled graphs. That is, we solve the ‘coupled isomorphism' problem separately for each frequent pattern discovered. We developed a novel approach for this step, which reduces the graph isomorphism problem to an item counting procedure of categorizing all subgraphs into isomorphic groups and counting the number of original graphs each group occurs. The method pipeline is illustrated in Fig. 1 (full details in the Supplementary Note 1, source code at http://zhoulab.usc.edu/struct2fun/).

### Spatial clusters constitute various regulatory communities

The discovery of frequent spatial cluster requires three parameters: the minimum size (that is, the number of chromatin domains in a cluster), the minimum edge density and the minimum occurrence frequency of the cluster in the genome structure population. Varying the three parameters, as expected, we observed that the number of identified frequent spatial clusters decreases with the increase of the minimum frequency threshold, the minimum edge density and the minimum size (details in Supplementary Note 2). In the following, we focus on the analysis of the set of 3,856 frequent spatial clusters discovered with the minimum size of 4, minimum edge density of 0.6 and minimum frequency of 1%. Our major conclusions are robust against the variation of the parameters (details in Supplementary Note 2).

Although local (intra-chromosomal) interactions are much more likely to occur than inter-chromosomal interactions, interestingly, 80.6% of the 3,856 clusters contained domains from multiple chromosomes. The majority of clusters contained domains from fewer than six chromosomes (Supplementary Fig. 3a). Intra-chromosomal clusters typically have higher frequency of occurrence in the structure population (on average 3,147 out of 10,000) than inter-chromosomal clusters (on average 509 out of 10,000). The distribution of the cluster sizes and frequencies is shown in Supplementary Fig. 3b. The relationships between sizes and frequencies for intra- and inter-chromosomal clusters are shown in Supplementary Fig. 3c,d respectively.

The 3,856 frequent spatial clusters, especially inter-chromosomal clusters, participate in a wide range of regulatory activities. We define a regulatory community as a frequent spatial cluster whose member domains are significantly co-enriched in binding to the same regulatory factor(s) (permutation test, *q*-value<0.05, see Supplementary Note 3). By analysing genome-wide binding data of 74 transcription factors^31^ of human lymphoblastoid cells, we found that 53.1% of all identified clusters constitute regulatory communities. Among those, 83 are *tRNA* synthesis communities due to their intense *RNAPIII*-binding signals; 3 are dominated by the polycomb binding proteins *YY1*; and 729 are transcription communities with significantly enriched *RNAPII*-binding signals. Remarkably, 707 (97%) of the transcription communities are inter-chromosomal. We also obtained DNA replication data^32^ of human lymphoblastoid cells to examine which clusters are enriched in early or late replication signals (details in Supplementary Note 3). We found that 333 clusters replicate at the beginning of S phase, whereas 4 clusters at the end of S phase.

Importantly, only a few of these regulatory communities can be inferred from the original Hi-C contact map. We ran two popular graph clustering algorithms, linkcomm^33^ and commDetNMF^34^, with different design principles, on the Hi-C contact map with a variety of different parameter settings in terms of edge cutoff, edge density, cluster size and algorithm-specific factors. From the linkcomm and commDetNMF algorithms in total, we obtained 3,157 and 15,755 clusters, which, however, show significant overlap (Jaccard Index>0.6) with only 12.5% and 14.1% of the 3,856 frequent spatial clusters, respectively. Furthermore, the frequent spatial clusters contained a much higher fraction (53.1%) of TF enriched clusters than those clusters directly derived from the Hi-C contact map (30.4% and 33.0% for clusters derived by linkcomm and commDetNMF, respectively). A closer examination of those clusters showed that >55% of clusters uniquely discovered by our approach, versus only 33.1% of the clusters uniquely discovered by linkcomm and 35.2% uniquely discovered by commDetNMF, are enriched in the TF binding. For details of the comprehensive comparison refer to Supplementary Note 4. The power of our approach can be explained by its use of chromatin interaction co-occurrence information in the modelled 3D genome population, which is entirely missing from the original Hi-C contact map. Such information facilitates the detection of subtle but biologically meaningful spatial clusters that are otherwise buried in the ensemble-averaged Hi-C data.

**Figure 2:**
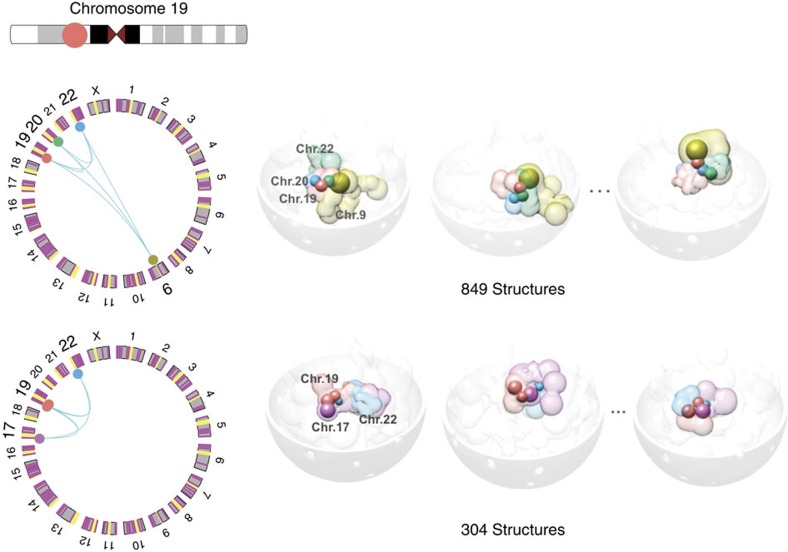
Functional plasticity of chromatin domain. An active domain in chromosome 19 can participate in two different clusters that are enriched with binding of the same transcription factors, including RNAPII, CTCF, NFYB and CREB1.

Interestingly, we observe that a chromatin domain can be part of multiple frequent clusters in different genome structures of the population. More importantly, domains tend to be shared by clusters that are regulated by the same group of transcription factors (Fisher's exact test, *P* value <10^−16^, Supplementary Table 1). Figure 2 illustrates that a transcriptionally active chromatin domain on chromosome 19 participates in two different inter-chromosomal clusters, which are both enriched with the same group of TFs, that is, RNAPII, CTCF, NFYB and CREB1. That is, regulatory communities of the same function can exchange their members. In other words, the structural plasticity of the human genome may far outweigh its functional plasticity, which also explains why the occurrence frequencies of individual regulatory communities are generally low.

**Figure 3:**
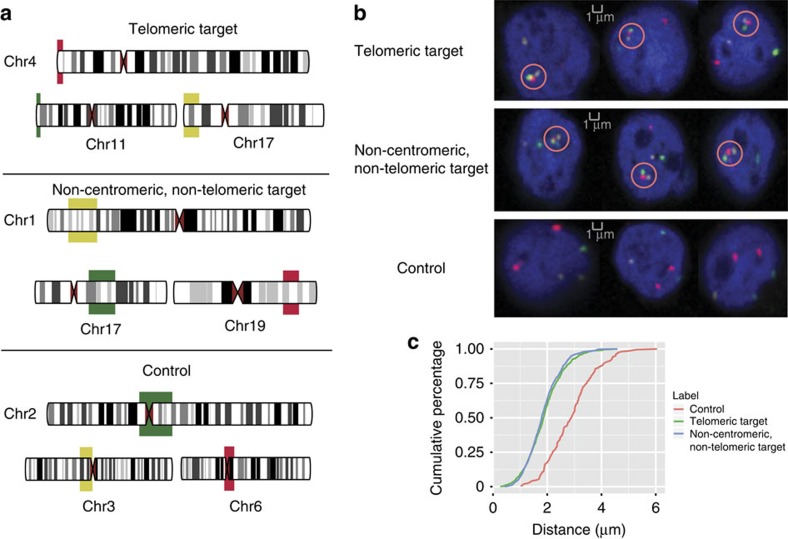
3D FISH experiments to validate the co-localization of domains within two inter-chromosomal clusters. (**a**) Layout of the 3D FISH experiments where the chromosomal locations of the clustered (targeted) regions and the control regions are shown. Telomeric targets were from p-arm telomeric regions of chromosome 4, 11 and 17. Non-centromeric, non-telomeric targets were from chromosomes 1, 17 and 19. Control regions were from sub-centromeric regions of chromosomes 2, 3 and 6. (**b**) Three example images of 3D FISH results in interphase nuclei are shown for telomeric target, non-centromeric, non-telomeric target and control regions, respectively. We used green, red, and yellow to label genomic locations of target and control regions. The chromosomal DNA was counterstained in blue with DAPI. Note that for the best view, the targeted region was the overlaid image of four channels (blue, green, yellow and red) from one of the exactly same Z-section, whereas the image of the control cell was the Z-projection of all z-sections from four channels. (**c**) Cumulative percentage of the average distances of the clustered targeted regions or the control regions were calculated from all the cells analysed (943 cells in telomeric targets, 982 cells in non-centromeric, non-telomeric targets and 595 cells in control regions). For two homologous regions of each chromosome, only one with the shortest distance from other chromosomes was counted and subject to analysis. In each cell, the distance (*x*-axis) was calculated as the average distance among three FISH probes.

To experimentally validate the co-localization of domains in identified clusters, we performed DNA 3D FISH on lymphoblastoid cells. We selected two clusters that each contains three domains from three different chromosomes and has enriched binding of RNAPII and other TFs. One cluster is formed by p-arm telomeric regions of chromosomes 4, 11 and 17; and the other is formed by domains far away from both centromeres and telomeres of the chromosomes 1, 17 and 19. As a comparison, we chose control regions on the centromeric locations of chromosomes 2, 3 and 6, which do not show co-localization from our modelled genome structures. (Figure 3a–c and Supplementary Table 2). Examining the average pairwise distances for domains in the predicted clusters and in the control regions, respectively, in 2,500 randomly chosen interphase nuclei, we confirmed that the domains in each of the 2 predicted clusters exhibit pronounced co-localizations (Fig. 3c).

### Centromeric domains are hubs for inter-chromosomal clusters

**Figure 4:**
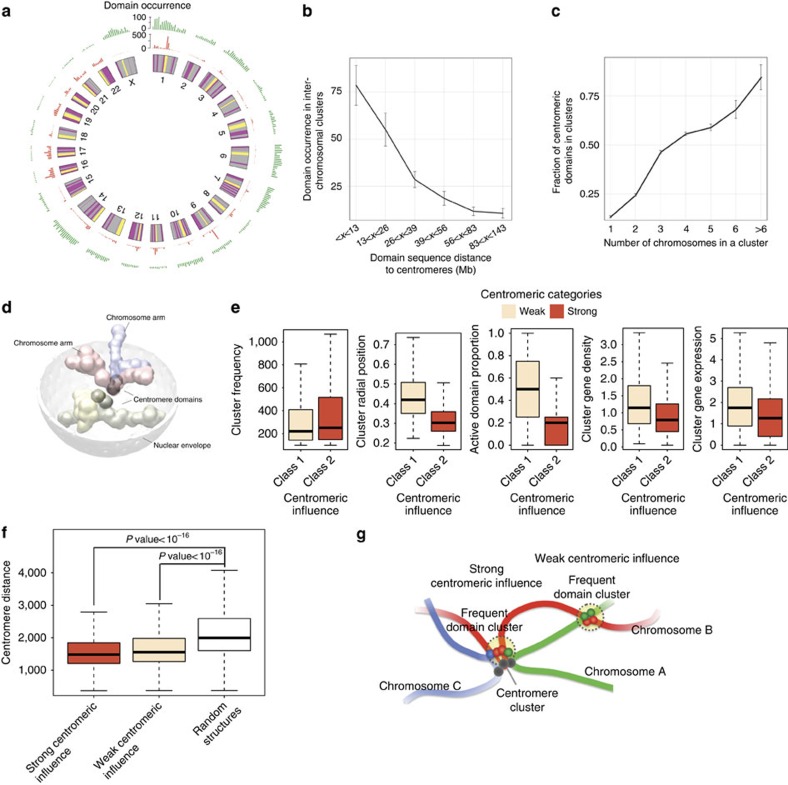
Centromeric influence on spatial clusters. (**a**) Domain occurrence in frequent spatial clusters. The full set of chromosomes is represented as the circular plot, where centromeric domains are coloured as yellow, active domains are coloured as red and inactive/other domains are coloured as grey. (**b**) A plot measuring the correlation between linear centromeric distance and domain occurrence in inter-chromosomal clusters. Data are shown as mean±s.d. of the mean. The number of domains in each group is 73, 72, 72, 73, 72 and 72. (**c**) A plot showing the correlation between the number of chromosomes in a cluster and the fraction of centromeric domains in the cluster. Data are shown as mean±s.d. of the mean. The number of clusters in each group is 749, 834, 1059, 972, 192, 36 and 14. (**d**) Illustration of centromere–centromere clustering. (**e**) Box plots comparing the characteristics of inter-chromosomal clusters with strong and weak centromeric influence, in terms of frequency, radial position, active domain proportion, gene density (the number of genes per 100 kb) and gene expression. (**f**) Box plots comparing centromere distance between three groups: clusters with strong centromeric influence, clusters with weak centromeric influence and clusters with weak centromeric influence that in random structures. (**g**) Illustration of inter-chromosomal clusters with strong and weak centromeric influence.

A striking observation is that among the 3,107 inter-chromosomal clusters, the vast majority (87%) contain at least 1 centromeric domain. Interestingly, centromeric domains of certain chromosomes as members of clusters are observed substantially more often than others. For example, the centromere domains of chromosomes 1 and 9 are involved in >500 inter-chromosomal clusters (Fig. 4a). In general, the closer a domain is to the centromere of its chromosome, the more frequently it participates in stable inter-chromosomal clusters (Fig. 4b). In addition, clusters involving more chromosomes generally have a higher proportion of centromeric domains (Fig. 4c). These observations suggest that centromeric domains are a major factor in chromosome intermingling.

Centromeres of different chromosomes tend to form clusters in human lymphoblastoid cells as shown by our recent structural modelling^17^ or advanced method to capture multiple-locus chromatin contacts^35^ on Hi-C data. Centromere–centromere colocalization could also be inferred from the Hi-C contact map (details in Supplementary Note 5). Our inspection of the 3D genome models revealed that when a centromere–centromere cluster forms, due to geometric constraints the centromeres are often located towards the central regions of the corresponding chromosome clusters, with chromosome arms emanating outward to constitute a ‘V-shaped' chromosome configuration (Fig. 4d). Consequently, the centromere regions are the crowded ‘meeting spots' of multiple chromosomes, therefore many inter-chromosomal chromosome clusters are formed in the proximity of centromeres. Importantly, the tendency to participate in centromere clusters varies widely among the chromosomes, which also explains why some inter-chromosomal clusters are formed more easily than others.

To further evaluate the centromeric influence on spatial genome organization, we classified the 3,107 inter-chromosomal clusters into 2 groups based on the proportion of centromeric domains in the cluster: 1,226 clusters contain between 0 and 30% centromeric domains (weak centromeric influence), and 1,881 clusters have a higher proportion (strong centromeric influence). We found that clusters with strong centromeric influence are generally more stable (occur with higher frequencies, Wilcox test *P* value=5.3 × 10^−5^), indicating that centromere–centromere interactions may play an important role in stabilizing inter-chromosomal clusters. We also found that clusters with strong centromeric influence are positioned closer to the nuclear centre, and have less gene density and lower gene expression level (all with the Wilcox test *P* values<10^−16^, Fig. 4e).

For inter-chromosomal clusters with weak centromeric influence, we further asked whether the involved centromeres are still co-localized even though centromere domains are not part of the frequent clusters. For each cluster, we calculated the average pairwise spatial distance between the centromeres of the chromosomes involved in the cluster. We compared three groups of centromere distances: clusters with strong centromeric influence, clusters with weak centromeric influence and randomly selected structures that do not contain the clusters with weak centromeric influence. We found that the average centromere distance are similar between the first two groups, and that both are significantly shorter than the last group (Fig. 4f). These results indicate that for inter-chromosomal clusters with a low portion of centromeric domains, the centromeres of the corresponding chromosomes are still co-localized, even if they are not part of the frequent cluster (Fig. 4g). Our results indicate that centromere–centromere clustering can be a major driving force for specific inter-chromosomal organization.

### Transcription factors may stabilize regulatory communities

Recent studies have shown that certain transcription factors, such as *Klf1*, *EKLF*, *GATA1* and *Nli/Ldb1*, can bridge long-range chromosomal contacts to form complexes of multiple co-regulated genes^36^,^37^,^38^,^39^,^40^. However, the extent and nature of this function is not clear. To examine the effect of TF binding in cluster stability, we computed the partial correlation between cluster frequency and the number of TFs with significantly enriched binding in the cluster, by removing the influence of centromeres on cluster frequency. We found a significant positive association (partial correlation of 0.19, *P* value=2.4 × 10^−26^, details in Supplementary Note 6). Indeed, for inter-chromosomal clusters under the same level of centromeric influence, those clusters bound by more TFs always have higher occurrence frequency (Supplementary Fig. 4). Our results indicate that transcription factors potentially stabilize inter-chromosomal contacts irrespective of the influence of centromeres.

**Figure 5:**
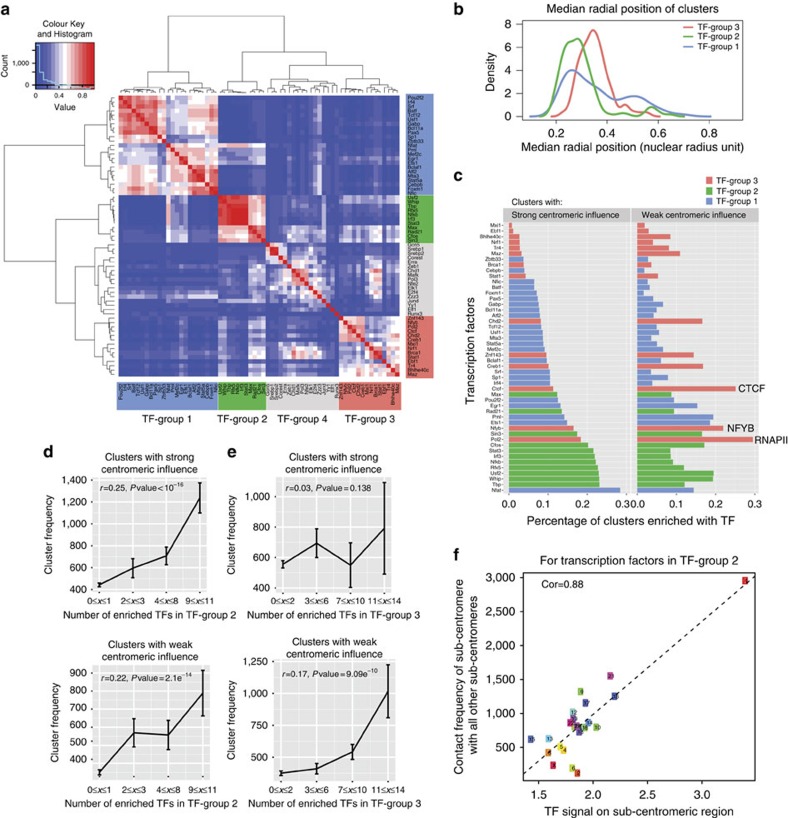
Transcription factors stabilization effects on spatial clusters. (**a**) About 65 TFs are classified into 4 groups based on their enrichment profiles across all inter-chromosomal clusters. (**b**) Radial position distributions of inter-chromosomal clusters exclusively enriched with individual TF-group. (**c**) Comparison of inter-chromosomal clusters with strong versus weak centromeric influence, in terms of the percentages of clusters enriched in TFs from different TF groups. (**d**) Correlation plot between cluster frequency and the number of enriched TFs in Group 2 with strong/weak centromeric influence. Data are shown as mean±s.d. of the mean. For clusters with strong centromeric influence, the number of clusters in each group is 339, 60, 230 and 220. For clusters with weak centromeric influence, the number of clusters in each group is 257, 69, 89 and 94. (**e**) Correlation plot between cluster frequency and the number of enriched TFs in Group 3 with strong/weak centromeric influence. Data are shown as mean±s.d. of the mean. For clusters with strong centromeric influence, the number of clusters in each group is 1,609; 207; 47 and 18. For clusters with weak centromeric influence, the number of clusters in each group is 958, 137, 94 and 37. (**f**) Correlation plot between the Group 2 TF signals on sub-centromeric regions and the subcentromere–subcentromere contact frequencies.

Moreover, the binding of TFs to chromatin clusters show functional-specific groupings, where four TF groups emerge based on their enrichment profiles across the chromatin clusters (Fig. 5a). The Group 1 is dominated by repressors, such as *PAX5*, *PML*, *MTA3* and so on, while Group 3 is dominated by many activators, such as *RNAPII*, *NFYB* and *EBF1*. TF-Group 2 is dominated by Immune Response TFs (IRTFs), including *Nf-KB*^41^, *c-Fos*^42^, *IRF3* (ref. 43), *STAT3* (ref. 44) and *RFX5* (ref. 45). Interestingly, the clusters enriched in these three groups of TFs showed different spatial distributions in the nucleus (Fig. 5b): clusters enriched with the TFs from the IRTF-dominated Group 2 are located most centrally in the nucleus; clusters enriched with the TFs from the activator-dominated Group 3 tend to be located between the nuclear centre and the periphery; and clusters with the TFs from the repressor-dominated Group 1 have a more dispersed radial distribution ranging across all positions.

The Group2 TFs (mainly IRTFs) are most enriched preferably in clusters with strong centromeric influences (Fig. 5c). Furthermore, the number of those TFs enriched in a cluster is significantly correlated with the cluster frequency, and such correlation is especially strong for clusters with strong centromeric influence (Fig. 5d). These observations lead to the hypothesis that this group of TFs may be closely associated with centromere clustering. Indeed, we found a positive correlation between the signal of these TFs in the sub-centromeric regions and the subcentromere–subcentromere contact frequencies (Fig. 5f, details in Supplementary Note 7). This evidence confirms the tight association between the IRTF binding and the centromere clustering, although it is still inconclusive whether IRTF stabilizes centromere clustering or centromere clustering stabilizes clusters bound by IRTFs.

On the other hand, only clusters with weak centromeric influence display a weak yet significant correlation between the cluster frequency and the number of enriched TFs in the TF-Group 3 (dominated by activators) (correlation of 0.17, *P* value=9.09 × 10^−10^, Fig. 5e). In addition, the proportion of clusters enriched with TF-Group 3 is 78% higher in clusters with weak centromeric influence compared with those with strong centromeric influence (Fig. 5c). Our results demonstrate that different factors impact the stability of regulatory communities at different nuclear locations. While centromere clustering and IRTF binding are tightly associated with the cluster stability in the nuclear centre, transcription activators (such as *RNAPII*, *CTCF* and *NFYB*) could potentially stabilize regulatory communities between the nuclear centre and the periphery.

### The genome structure population contains multiple substates

**Figure 6:**
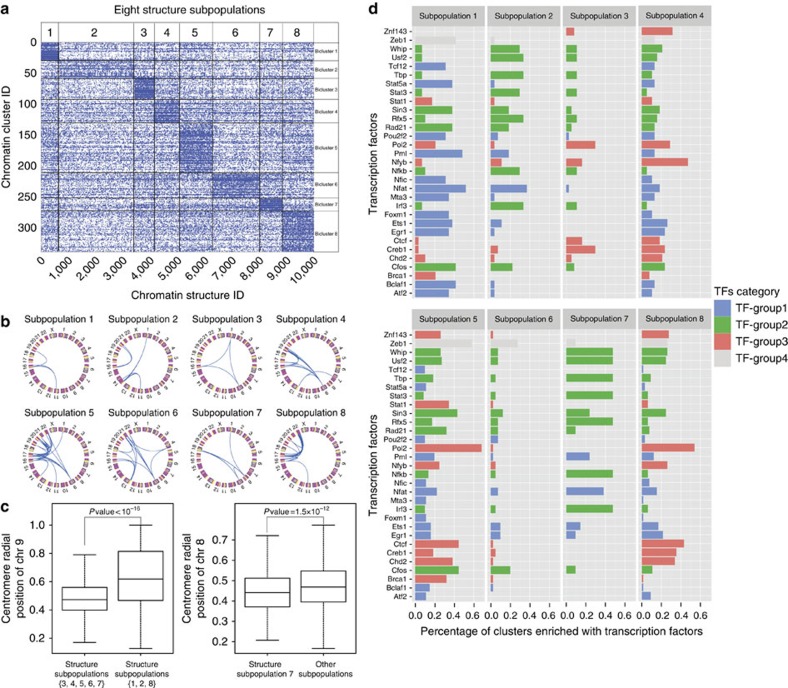
Genome structure subpopulation analysis. (**a**) 10,000 structures are partitioned into 8 non-overlapping subpopulations. (**b**) Each structure subpopulation has a distinct pattern of inter-chromosomal contacts, which display the top 20% high-frequency domain pairs. (**c**) Compare the radial position of specific chromosomes in different structure subpopulations. (**d**) The eight structure subpopulations have different top-enriched TFs.

The detection of distinct regulatory communities incites us to study the co-occurrence or mutual exclusivity of these communities in a single genome structure. To achieve this, we performed biclustering analysis of the occurrence profiles of all clusters across the population. We identified eight non-overlapping subsets of genome structures spanning the whole structure population (Fig. 6a), where each subset is characterized by the co-occurrence of a set of specific spatial clusters. In other words, we were able to divide the structures in the population into different structural states according to the presence or absence of spatial clusters. Note that using domain contacts as features would lead to very different structure subpopulations (details in Supplementary Note 8).

The eight structure subpopulations differ significantly in their inter-chromosomal domain–domain contact maps (Fig. 6b). For example, centromere of chromosome 9 have high-frequency contacts with other chromosomes only in subpopulations 3, 4, 5, 6 and 7. After investigating the 3D models, we found that the centromeres of chromosome 9 have significantly smaller radial positions in these subpopulations (Fig. 6c), giving its chromosomes more chance to intermingle with other specific chromosomes. Analogously, centromere of chromosome 8 displays high-frequency chromatin contacts only in subpopulation 7 (Fig. 6b), where its radial position is located more towards nuclear interior (Fig. 6c).

Interestingly, the identities of the most enriched TFs are quite different in the clusters of the different subpopulations (Fig. 6d). For example, among the 31 top-enriched TFs, 70% showed significant signal difference in the clusters of subpopulations 1 and 2 (Wilcox test *P* value <0.05). In subpopulation 1 the most enriched TFs are from the Group 1 (mainly repressors). In subpopulations 2 and 7, Group2 (mainly IRTFs) are dominant, while in subpopulations 3, 4, 5 and 8 the most enriched TFs are from Group 3 (mainly activators). Our results suggest that the regulation of certain transcription factors may be facilitated in individual cell states, which, in turn, may stabilize specific regulatory communities. It is known that many actively transcribed genes are only expressed in a portion of the cell population^15^,^16^,^46^,^47^,^48^,^49^. Our study indicates that the diversity of genome structures may contribute to the diversity of expression in an isogenic cell population.

## Discussion

We present a graph-based computational framework for the analysis of 3D genome structure populations, for which traditional structural analysis tools are not suitable due to the highly plastic nature of genome structures. Our method can comprehensively identify frequently occurring chromatin clusters in tens of thousands of 3D genome structures. Integrating epigenomics and functional genomics data, we show that the clusters are involved in a wide range of nuclear processes, with transcription being most prevalent. Strikingly, the majority of these clusters are not detectable in the Hi-C contact map. Interestingly, we found that a chromatin domain can belong to different transcription communities in different genome structures; however, these communities tend to be regulated by same transcription factors. This observation reveals the functional degeneracy of the highly plastic human genome structures.

We identified two major factors, centromere clustering and transcription factor binding, that significantly contribute to the stability of the 3D regulatory communities in lymphoblastoid cells. Centromere clustering creates crowded ‘meeting spots' of multiple chromosomes in the nuclear centre, facilitating the formation of inter-chromosomal contacts and hence functional chromatin clusters. Note that the impact of centromere clustering reaches far beyond the nuclear centre. Even among chromatin clusters containing a low percentage of centromere domains, the respective centromeres are still co-localized. In this case centromeres serve as anchors, and active domains nearby loop outward and intermingle, potentially creating hotspots to form transcriptional communities. We further show that binding of specific transcription activators could potentially stabilize such communities, particularly between the nuclear centre and the periphery. Another key finding is that the localization of sets of TFs into transcription communities demonstrate specific patterns, in particular, activators and repressors are grouped into separate transcriptional communities. In other words, we discovered that combinatorial regulation of TFs, until now only discussed in the context of linear genome sequences, in fact also exists in the 3D space. We note that our major findings are not sensitive to the parameter settings for the frequent clustering mining algorithm (see Supplementary Note 2).

Using the frequently occurring chromatin clusters as structure features, we were able to group the 10,000 highly variable 3D genome structures into 8 groups (substates) exhibiting similar feature profiles. We show that the transcriptional communities present in the genome structures differ dramatically from substate to substate, indicating that 3D genome structures may contribute to expression variability across cells.

Our method is generally applicable to genome structures at any resolution. This paper exemplifies the utility of our method at the resolution of macrodomains. Intuitively, one would hope that the unit of resolution (for example, macrodomains) shall not vary across the genome population. Recent single-cell Hi-C experiments showed that topological domains are largely conserved across cells within a sample^50^. As a realistic approximation, we assume that macrodomains possess the same property. As the 3D genome modelling quickly approaches the resolution of topological domains, more functional and regulatory inferences can be derived.

Our method is also applicable to the functional analysis of a collection of single-cell Hi-C data, although currently single-cell Hi-C technology is still hampered by its very limited capture efficiency and the cost bottleneck to cover the highly variable genome conformation space. Therefore, our strategy of frequent pattern discovery from a population of 3D genome structures, deconvoluted from the ensemble-averaged Hi-C data, presents a realistic and powerful approach to perform structure-function mapping in 3D genomes.

## Methods

### Data pre-processing

We performed the TCC experiment in human lymphoblastoid cells. Together with data for the same cell type generated in a previous study^3^, we analysed ∼50 million read pairs of deep-sequencing data. The data pre-processing and normalization were described in our previous work^3^. The filtered pairwise fragment reads were aggregated in a genome-wide interaction matrix using 4,992 bins. For details of the population-based genome structure modelling method, see our previous paper^3^.

### 3D DNA FISH

The DNA FISH probes were synthesized by Empire Genomics Inc (for the detailed information about the chromosomal location and fluorescence labelling of the probes, see Supplementary Table 2). Human lymphoid cell line GM12878 cells were fixed and permeablized following the protocols^3^,^51^ with slight modification. The denaturation and hybridization steps were performed according to the protocols suggested by the manufacturer. Three probes (150 ng each) for either targeted regions or control regions were mixed thoroughly with 18 μl hybridization buffer (provided by the manufacturer) and applied evenly with the sample on a microscope slide. After hybridization, the samples were washed several times to remove unbound FISH probes, and mounted on microscope slides with 10 μl DAPI mounting solution for each slide. The FISH images were acquired with Zeiss Laser Scanning Confocal microscope (LSM-780) with × 63 oil immersion objective lenses. Optical sections (Z stacks) with 0.25–0.5 μm apart were obtained with alternative scanning (frame mode) of two lasers each, and stored in four separate channels with the ZEN software provided by the manufacturer. The FISH results were analysed with ImageJ^52^ and Nemo^53^.

### Partitioning spatial genome structures into subpopulations

For each inter-chromosomal cluster, we constructed a binary vector of length *N* storing its occurrence in the *N* structures. This vector can be interpreted as an activity profile of the cluster in different structures. We combined these vectors into the cluster co-occurrence matrix *C*=(*c*_*ij*_)_*M* × *N*_, where *c*_*ij*_=1 when cluster *i* occurs in structure *j*, otherwise *c*_*ij*_=0. We are interested in the co-occurrence pattern, so called ‘bicluster', consisting of a subset of clusters and structures, such that the member clusters are more likely to co-occur within the bicluster than those outside of the bicluster. We applied a non-negative matrix factorization (NMF)-based biclustering algorithm^54^ to discover the biclusters. We first removed 347 inter-chromosomal clusters with the occurrence frequency ≥1,000 from the matrix *C*, because they have prevalent occurrences across the population and thus cannot provide useful information. Then we performed the NMF-based biclustering algorithm, resulting in eight biclusters.

## Additional information

**How to cite this article:** Dai, C. *et al*. Mining 3D genome structure populations identifies major factors governing the stability of regulatory communities. *Nat. Commun.* 7:11549 doi: 10.1038/ncomms11549 (2016).

## Supplementary Material

Supplementary Information Supplementary Figures 1-11, Supplementary Tables 1-9, Supplementary Notes 1-8 and Supplementary References
