# Supplementary material for: Mining 3D genome structure populations identifies major factors governing the stability of regulatory communities
Source: Nat Commun. 2016 May 31;7:11549. doi: 10.1038/ncomms11549 (PMC4895025; doi:10.1038/ncomms11549)
Supplement: Supplementary Information — Supplementary Figures 1-11, Supplementary Tables 1-9, Supplementary Notes 1-8 and Supplementary References [file ncomms11549-s1.pdf]

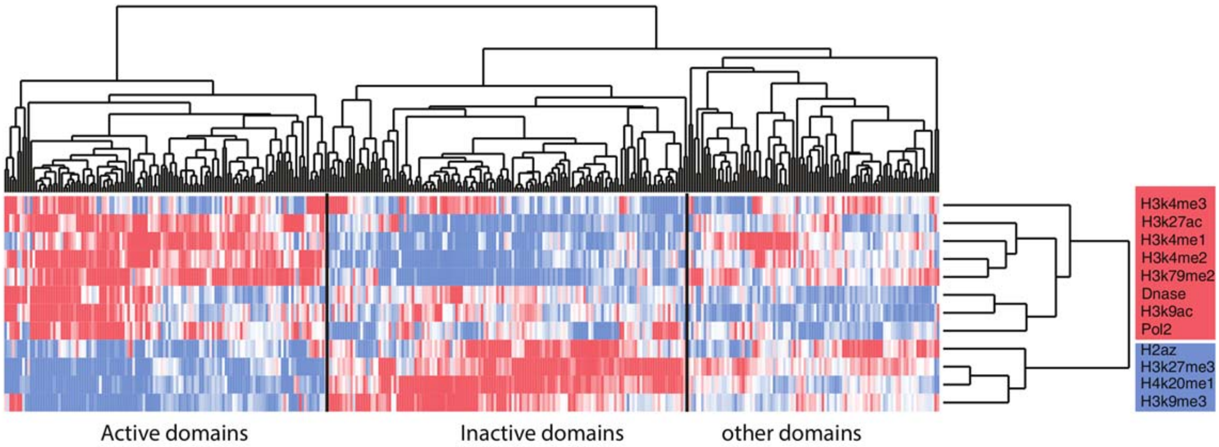

Supplementary Figure 1: Hierarchical clustering of 365 non-centromeric domains into active, inactive, and others, based on 12 epigenetic/transcriptional marks.

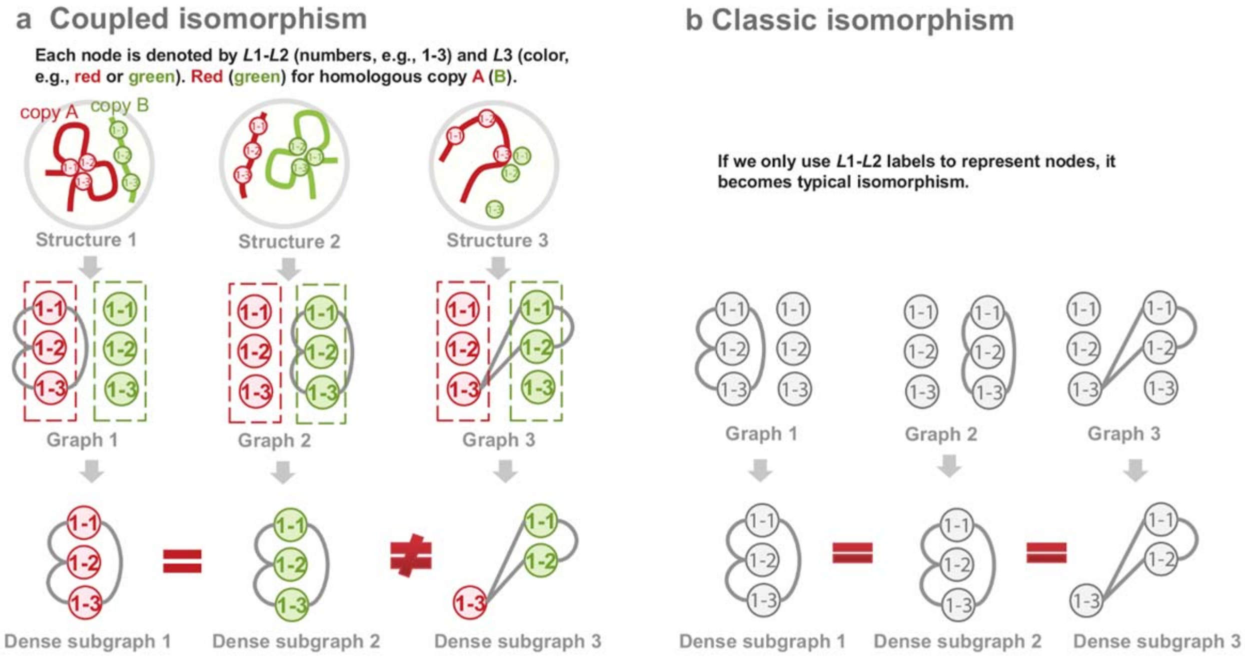

Supplementary Figure 2: Illustration of the difference between the coupled isomorphism (the unique feature of our problem) and the classic isomorphism. This example demonstrates the unique feature of our problem. Chromosome 1 has two copies: copy A (red) and copy B (green). In this example, we have three structures and each domain copy is labeled by *L*1-*L*2 (chromosome index and domain index, represented by two numbers) and *L*3 (homologous copy index, represented by node color). It is worth noting that we do not know whether copy A in a structure corresponds to copy A in other structures; therefore A (red) and B (green) are only used for distinguishing two copies within a structure, and does not have mapping relationships with other structures. (a) Coupled isomorphism in our problem. Each structure is first transformed into a chromatin interaction graph (*CIG)* where each node is labeled by *L*1-*L*2 with color *L*3. Then we identified a dense subgraph from each *CIG*. It can be seen that the dense subgraph 1 (i.e., {1-1-A, 1-2-A, 1-3-A}) equals to the dense subgraph 2 (i.e., {1-1-B, 1-2-B, 1-3-B}), because all three domains are from the same chromosome copy and all the chromatin interactions are *cis* (shown in Structures 1 and 2). However, the dense subgraph 3 (i.e., {1-1-B, 1-2-B, 1-3-A}) consists of domains from different copies of chromosome 1 and therefore has one *cis* interaction and two *trans* interactions. Although all three nodes are from the same domains (i.e., the same *L*1-*L*2 labels) as those in the dense subgraphs 1 and 2, three structures show that the dense subgraph 3 should not equal to the dense subgraphs1 and 2. (b) Classic isomorphism. If we only use *L*1 and *L*2 labels for the three structures (removing *L*3, i.e. node color), two homologous copies of any domain will have the same labels. Therefore, three dense subgraphs identified in three graphs are equivalent to each other.

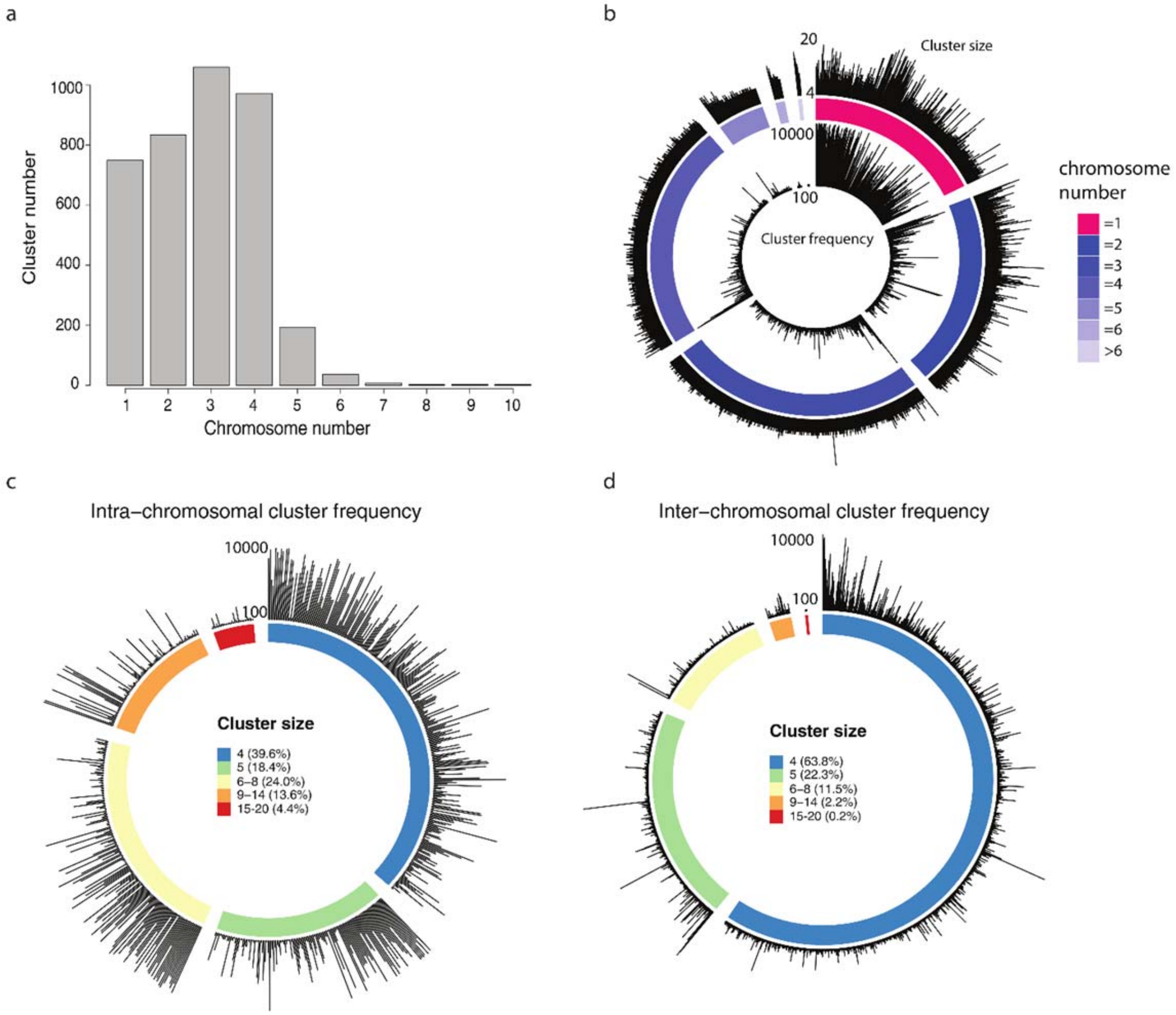

Supplementary Figure 3: Basic statistics of spatial clusters. (a) Histogram of spatial cluster number per the number of chromosomes in the cluster. (b) The distribution of spatial cluster size and frequency per the number of chromosomes in the cluster. (c) The distribution of intra-chromosomal cluster frequency per cluster size. (d) The distribution of inter-chromosomal cluster frequency per cluster size.

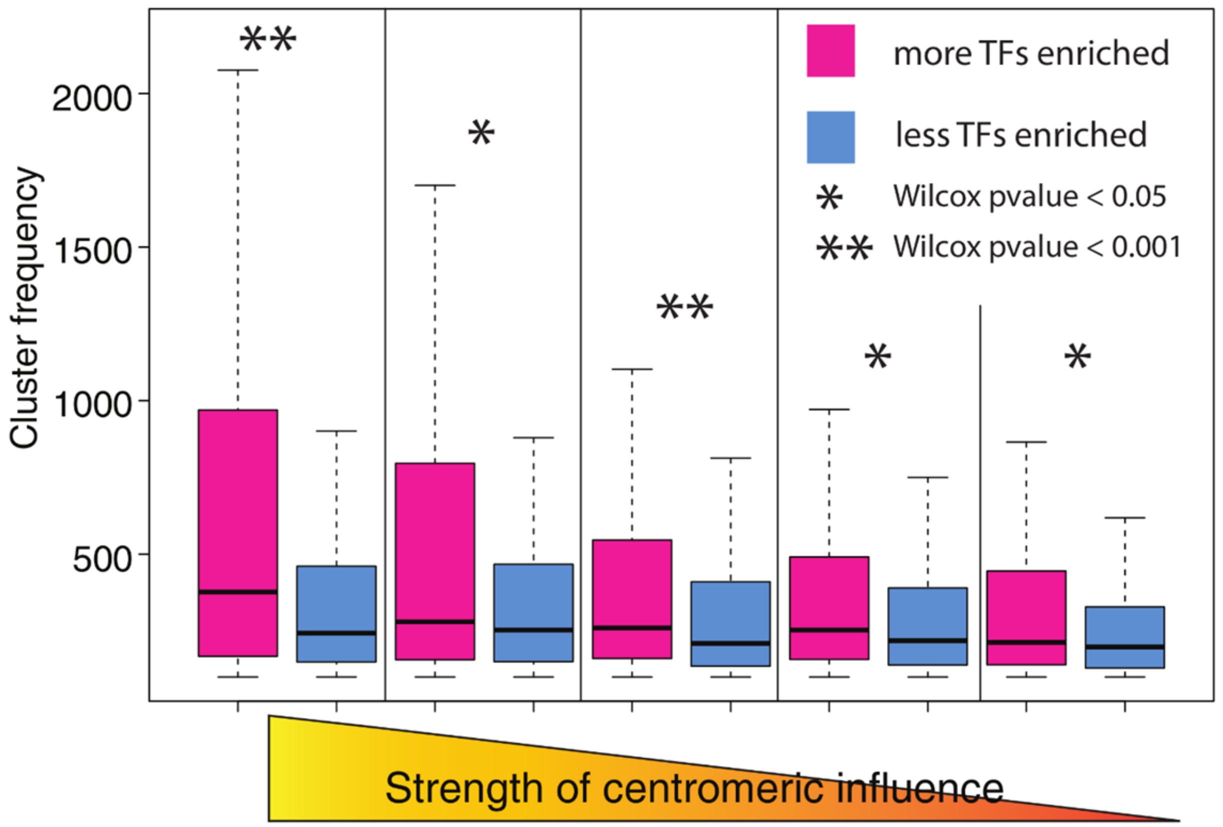

Supplementary Figure 4: TFs stabilize inter-chromosomal clusters with different centromeric influence. Inter-chromosomal clusters are partitioned into five different groups, in the decreasing order of the proportion of centromeric domains in the cluster. The numbers of grouped inter-chromosomal clusters are 221, 642, 801, 785, and 658, corresponding to centromeric domain proportions 80%-100%, 60%-80%, 40%-60%, 20%-40%, 0-20%. In each group, clusters are sorted based on the number of TFs enriched in the cluster, and further split into two halves, such that one half has more TFs enriched, and the other half has less TFs enriched, then we compare cluster frequency between the two halves.

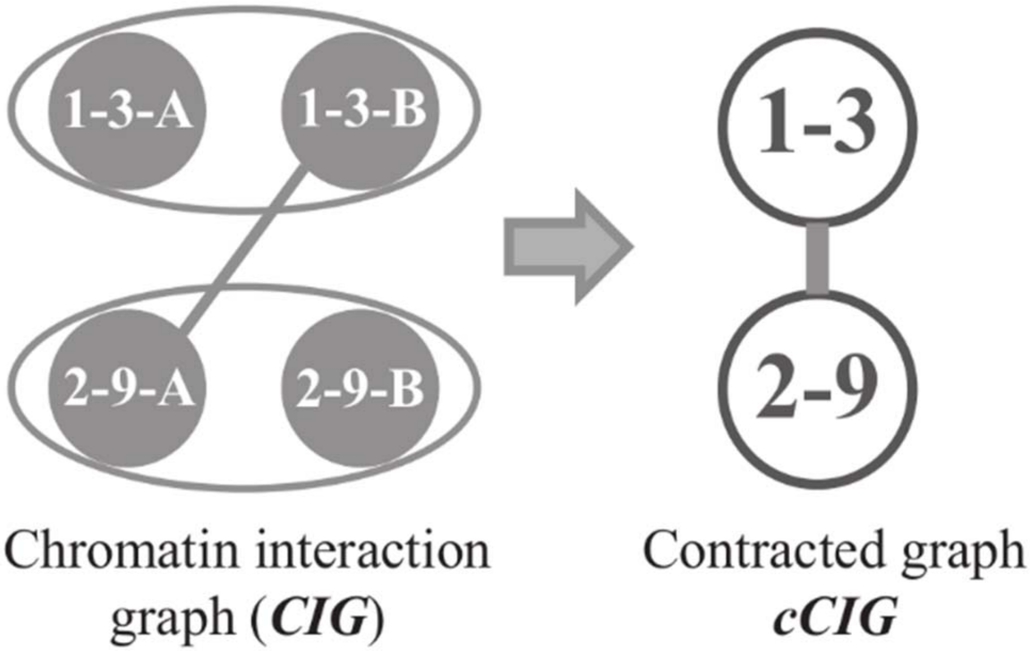

Supplementary Figure 5: Node contraction procedure of transforming a chromatin interaction graph (*CIG*) into a contracted graph (*cCIG*). Two contracted nodes in *cCIG* should have an edge, if there exists at least one edge between their corresponding nodes in *CIG*.

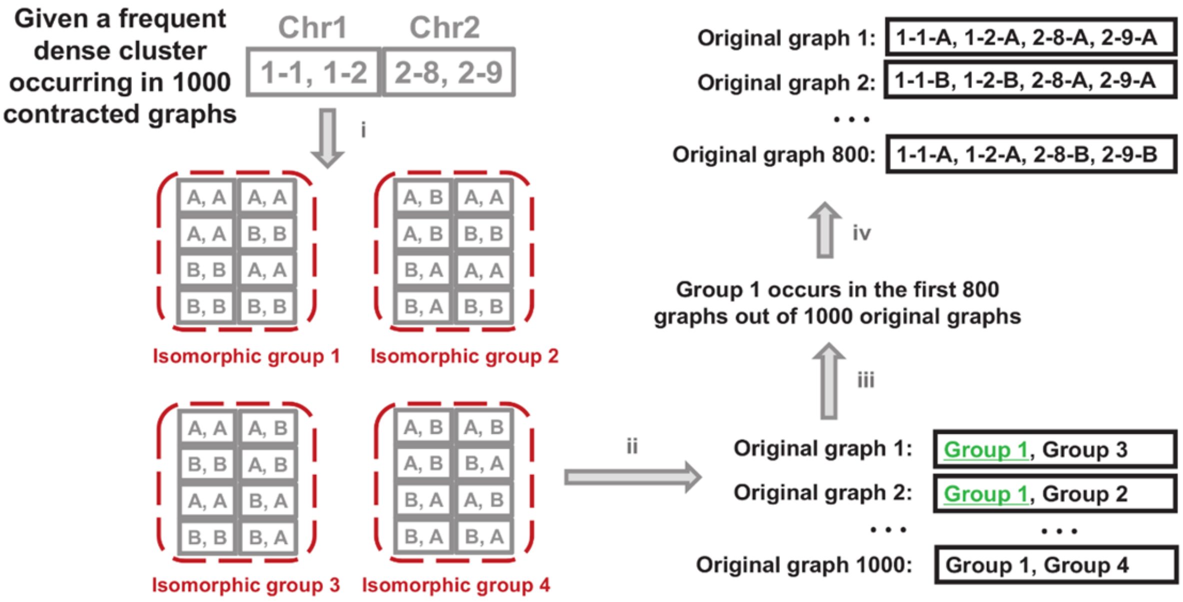

Supplementary Figure 6: Illustration of the counting algorithm flowchart for pattern recover problem. Given a frequent dense cluster that occurs in many contracted graphs, this algorithm aims to recover their counterparts in the original graphs. For example, a cluster consists of four domains (located in chromosome 1 and chromosome 2) and occurs in 1000 contracted graphs. The flowchart of the recover procedure is shown in four steps: (i) Expand the contracted cluster to 2^N=16 subgraphs with specific domain copy information (copy A or B), where *N* is the number of domains in the cluster. All subgraphs are categorized into four isomorphic groups and each group represents one type of allele-specific configuration of this cluster. For example, isomorphic group 1 means “all homologous domains of chromosome 1 are from the same copy, and all homologous domains of chromosome 2 are also from the same copy.” (ii) Select the isomorphic groups which have at least one subgraphs whose densities ≥ a threshold (e.g., 0.6) in each original graph. (iii) Count the frequency of each group and determine the most frequent group. The frequency of an isomorphic group is the number of original graphs this group occurs. So the most frequent group is the one that occurs in most original graphs. For example, the isomorphic group 1 occurs in the first 800 graphs out 1000 graphs and is considered as the most frequent group (highlighted in green). (iv) Extract dense subgraphs of the most frequent group from each original graph it occurs. We extract a dense subgraph of group 1 for each original graph where group 1 occurs. These extracted dense subgraphs are exactly the recovered patterns in the original graphs – the output of the pattern recovering procedure.

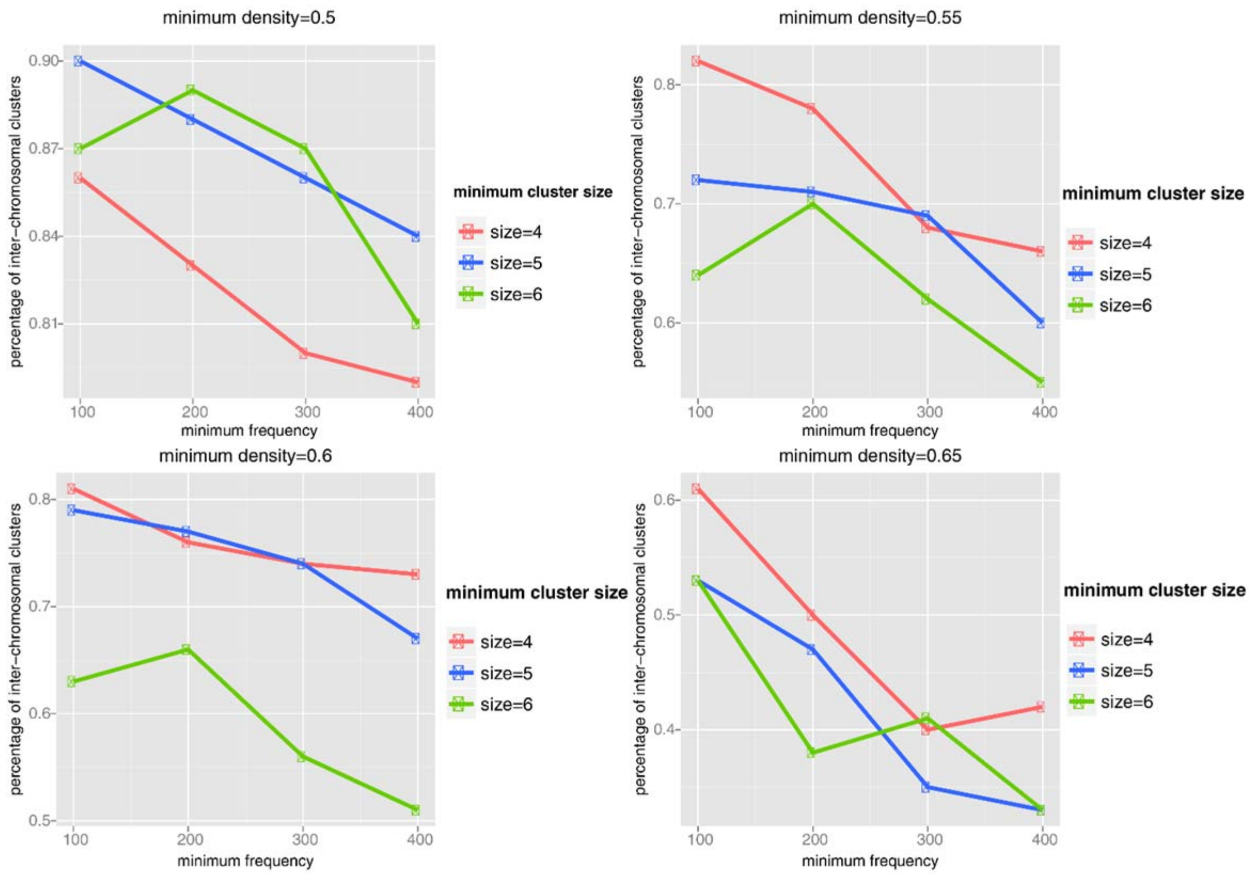

Supplementary Figure 7: The percentage of inter-chromosomal clusters generally decreases as the minimum cluster size, the minimum cluster density, and the minimum cluster frequency increase.

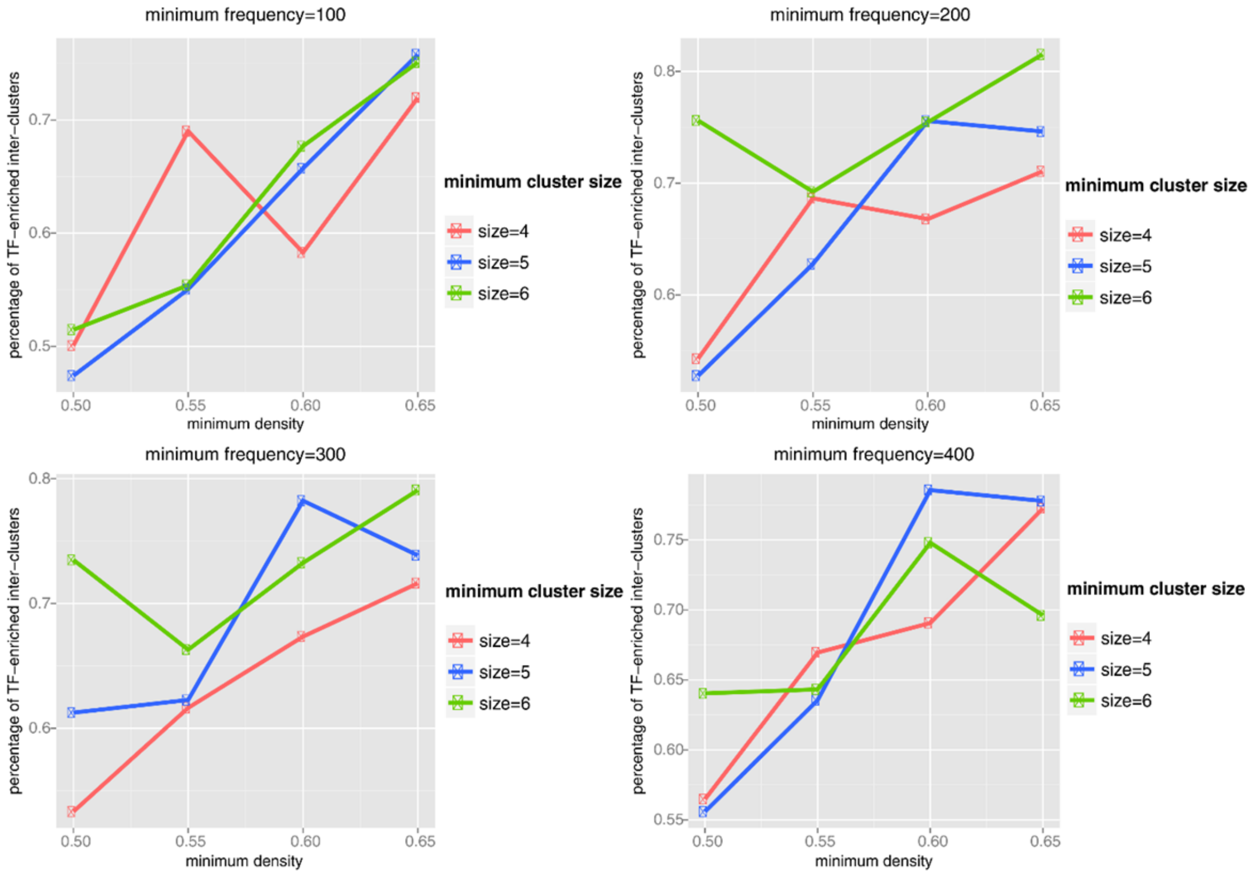

Supplementary Figure 8: The percentage of TF-enriched inter-chromosomal clusters generally increases as the minimum cluster density and the minimum cluster frequency increase.

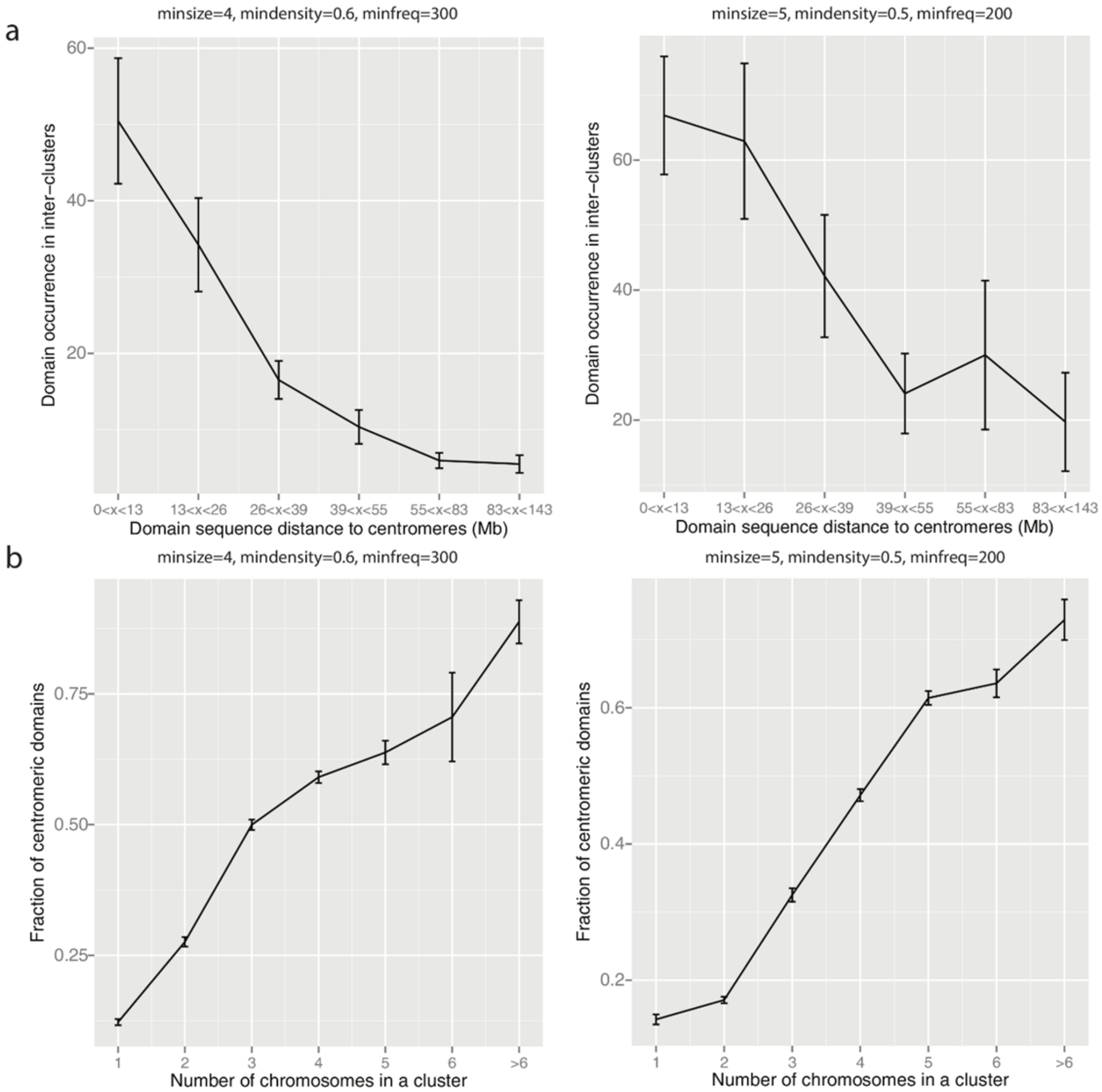

Supplementary Figure 9: Centromeric domains are likely to serve as hubs for inter-chromosomal clusters. (a) The frequency of a domain to participate in inter-chromosomal clusters increases as the distance between the domain and the centromere of the respective chromosome decreases, shown under two different parameter settings. (b) The number of chromosomes in a cluster and the fraction of centromeric domains in the cluster show positive correlation under two different parameter settings.

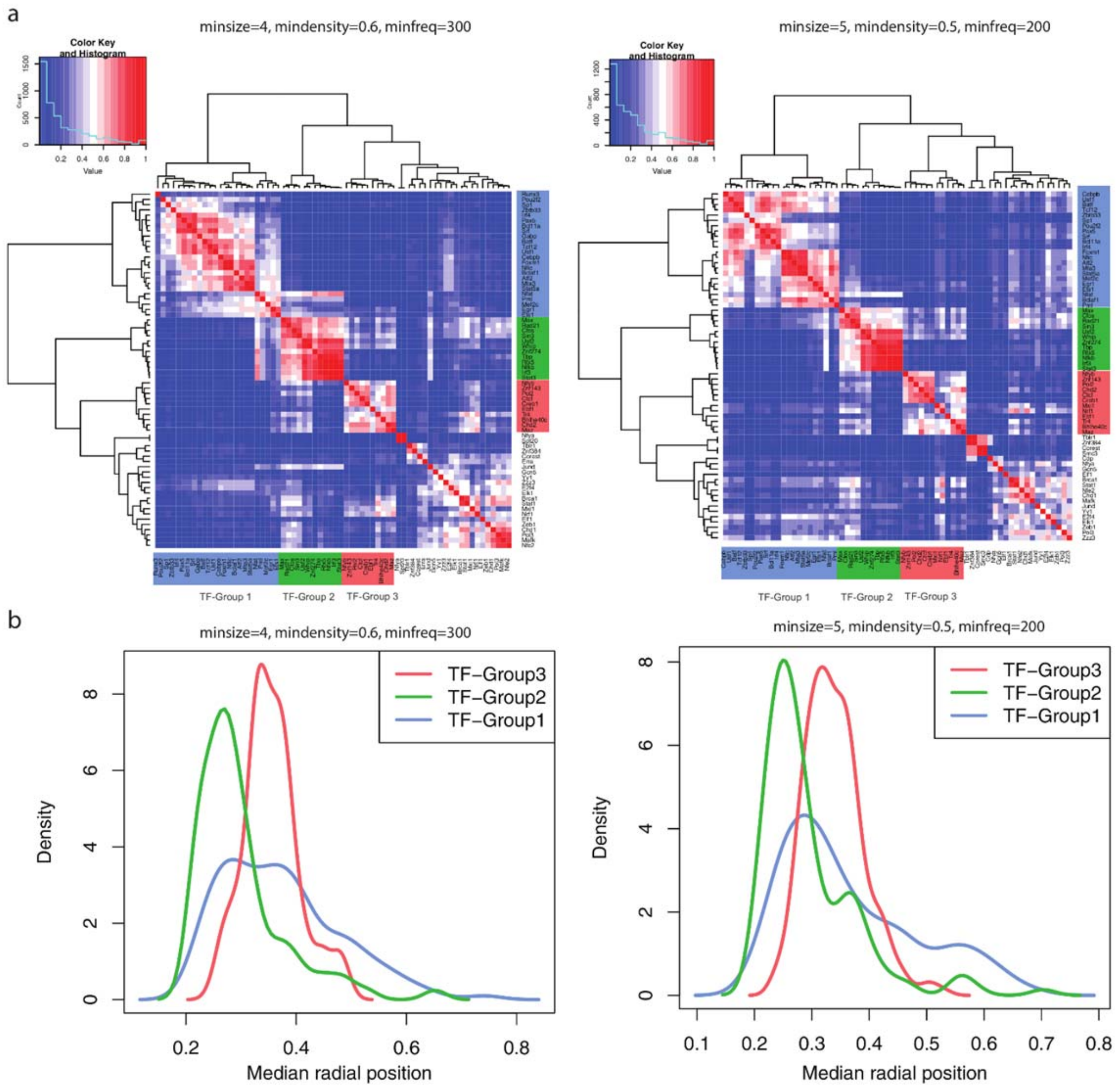

Supplementary Figure 10: Transcription factor organization in inter-chromosomal spatial clusters. (a) TF clustering results based on their enrichment profiles across all inter-chromosomal clusters, shown under two different parameter settings. (b) Radial position distributions of inter-chromosomal clusters exclusively enriched with individual TF groups, shown under two different parameter settings.

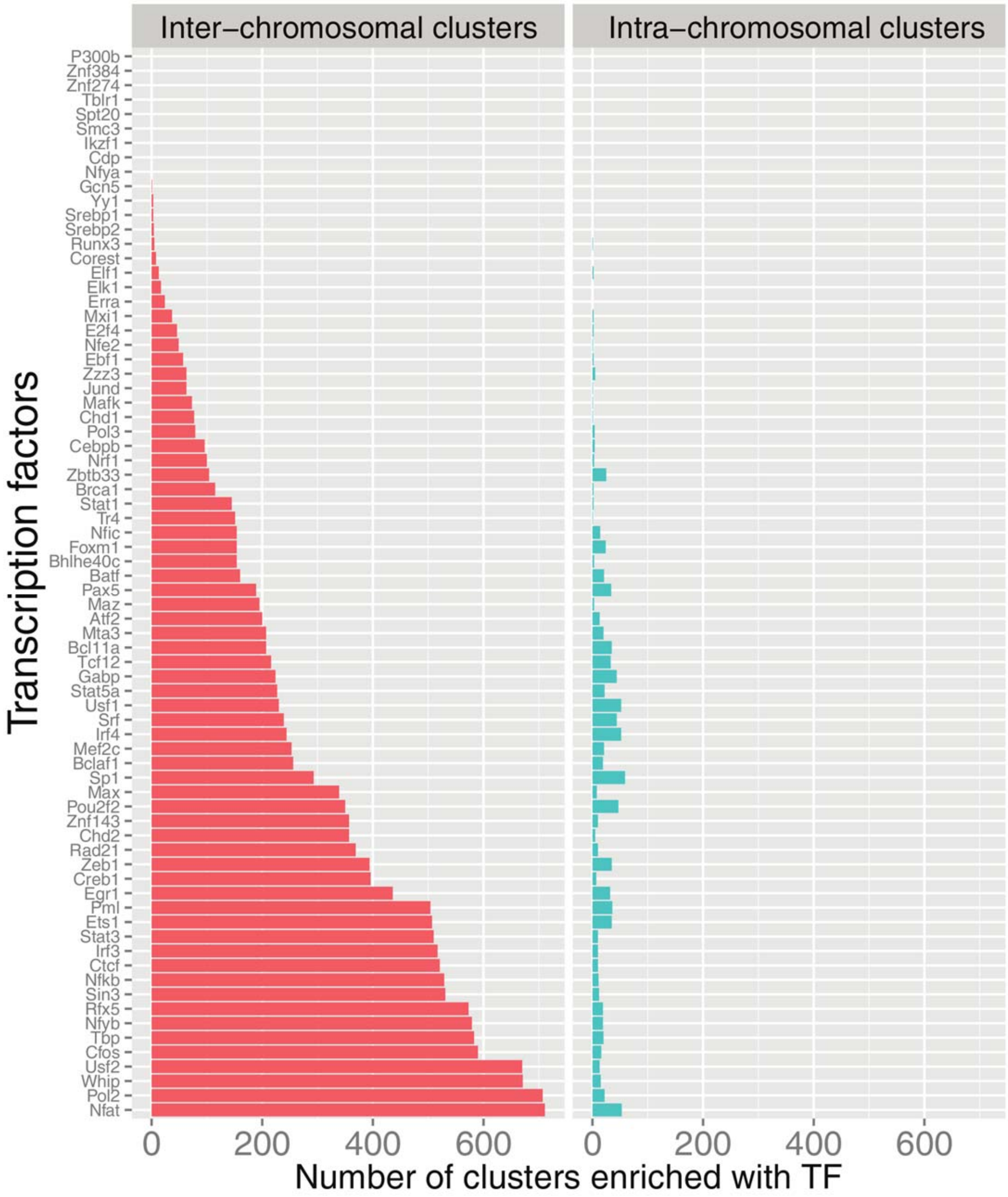

Supplementary Figure 11: The number of inter-/intra- chromosomal clusters enriched with each TF.

	Clusters enriched with the same TFs	Clusters enriched with different TFs
Clusters that share domains	5466	17995
Clusters that don't share domains	2204	67529

Supplementary Table 1: Fisher's exact test table indicates that domains tend to be shared by clusters that are regulated by the same transcription factors.

Domain	Chromosomal location	Probe name (BAC clone)	Probe localization	Probe color (Fluorophore)
**Telomeric targeted probes**				
Domain 75	Chr4: 10000-4433960	RP11-875G10	chr4:1706259-1883327	Red
Domain 226	chr11:60000-3973178	RP11-1007G14	chr11:378238-567259	Green
Domain 334	Chr17: 0-8987390	RP11-818O24	chr17:1153804-1350347	Yellow
**Non centromeric, non-telomeric probes**				
Domain 4	chr1:19737856-47067667	RP11-401H24	chr1: 27883545-28058422	Yellow
Domain 340	Chr17: 33144995-49419943	RP11-782E1	chr17: 43983233-44203399	Green
Domain 370	Chr19: 45242424-51834512	RP11-902P17	chr19: 46042361-46250605	Red
**Control probes**				
Domain 40	chr2:84589799-114915216	RP11-831B17	chr2: 96977476-97209012	Green
Domain 58	Chr3: 73391554-90504854	RP11-1082I19	chr3: 87569564-87784200	Yellow
Domain 128	Chr6: 57153390-70308348	RP11-973P24	chr6:65112008-65298120	Red

Supplementary Table 2: Detailed information of FISH probes used in the experiments.

TF	# times in top enriched TF list	TF	# times in top enriched TF list
c-FOS	48	RNAPII	47
NFYB	48	NF-kB	46
RFX5	48	SIN3	46
USF2	48	TBP	46
WHIP	48	IRF3	45

Supplementary Table 3: Top enriched transcription factors in inter-chromosomal clusters for all the 48 different parameter combinations.

	Number of all map-based clusters	TF enriched map-based clusters %	unique freq. spatial cluster		common cluster		unique map-based clusters	
			%	TF enriched %	%	TF enriched %	%	TF enriched %
linkcomm	3157	30.4%	87.5%	55.2%	12.5%	38.8%	69.7%	33.1%
commDetNMF	15755	33.0%	85.9%	55.8%	14.1%	37.2%	74.3%	35.2%
Note	$\lvert B \rvert$	$\frac{\lvert \text{TF enriched } B \rvert}{\lvert \text{B} \rvert}$	$\frac{\lvert \text{unique } A \rvert}{\lvert A \rvert}$	$\frac{\lvert \text{TF enriched } A \rvert}{\lvert \text{unique } A \rvert}$	$\frac{\lvert A \cap B \rvert}{\lvert \text{A} \rvert}$	$\frac{\lvert \text{TF enriched } A \cap B \rvert}{\lvert A \cap B \rvert}$	$\frac{\lvert \text{unique } B \rvert}{\lvert B \rvert}$	$\frac{\lvert \text{TF enriched } B \rvert}{\lvert \text{unique } B \rvert}$

Supplementary Table 4: Overlap and TF Enrichment comparison between the frequent spatial clusters and map-based clusters with the overlap definition of $J(a, b) \geq 0.6$. In this table, $A = \{a_1, \ldots, a_{3856}\}$ denotes the set of 3856 frequent spatial clusters and $B = \{b_1, b_2, \ldots\}$ denotes all the map-based clusters that are the union of all cluster sets identified by different parameters of a particular algorithm. "$\text{TF enriched } A$" ("$\text{TF enriched } B$") is the subset of frequent spatial clusters (map-based clusters) that are enriched with the binding of TFs. "$\text{TF enriched } A \cap B$" is the subset of frequent spatial clusters that not only overlap with *B* but also are enriched with the binding of TFs. "$\text{unique } A$" is the subset of frequent spatial clusters that don't have overlap with map-based clusters. "$\text{unique } B$" is the subset of map-based clusters that don't have overlap with frequent spatial clusters.

	Number of all map-based clusters	TF enriched map-based clusters %	unique freq. spatial cluster		common cluster		unique map-based clusters	
			%	TF enriched %	%	TF enriched %	%	TF enriched %
linkcomm	3157	30.4%	99.53%	53.3%	0.47%	27.8%	99.3%	30.5%
commDetNMF	15755	33.0%	99.22%	53.3%	0.78%	30.0%	99.7%	33.0%
Note:	$\|B\|$	$\frac{\|\text{TF enriched } B\|}{\|\text{B}\|}$	$\frac{\|\text{unique } A\|}{\|A\|}$	$\frac{\|\text{TF enriched } A\|}{\|\text{unique } A\|}$	$\frac{\|A \cap B\|}{\|\text{A}\|}$	$\frac{\|\text{TF enriched } A \cap B\|}{\|A \cap B\|}$	$\frac{\|\text{unique } B\|}{\|B\|}$	$\frac{\|\text{TF enriched } B\|}{\|\text{unique } B\|}$

Supplementary Table 5: Overlap and TF Enrichment comparison between the frequent spatial clusters and map-based clusters with the overlap definition of $J(a, b) \geq 0.9$. In this table, $A = \{a_1, \dots, a_{3856}\}$ denotes the set of 3856 frequent spatial clusters and $B = \{b_1, b_2, \dots\}$ denotes all the map-based clusters that are the union of all cluster sets identified by different parameters of a particular algorithm. “$\text{TF enriched } A$” (“$\text{TF enriched } B$”) is the subset of frequent spatial clusters (map-based clusters) that are enriched with the binding of TFs. “$\text{TF enriched } A \cap B$” is the subset of frequent spatial clusters that not only overlap with *B* but also are enriched with the binding of TFs. “$\text{unique } A$” is the subset of frequent spatial clusters that don’t have overlap with map-based clusters. “$\text{unique } B$” is the subset of map-based clusters that don’t have overlap with frequent spatial clusters.

FDR-adjusted p-value cutoff	0.05	0.01	0.005
Colocalization frequency of triplet chromsomes	78.5%	51.8%	43.5%

Supplementary Table 6: Subcentromeric regions have significant colocalization signal from Hi-C data

	Our method	RB	RBR	BAGGLO
Our method	1			
RB	0.00581	1		
RBR	0.00354	0.44552	1	
BAGGLO	0.00363	0.47486	0.27943	1

Supplementary Table 7: Normalized mutual information (NMI) measure to evaluate how similar the population substates obtained by different methods. The higher NMI is, the more similar two methods' clustering results are. Note that RB stands for repeated bisecting K-means method, RBR stands for refined RB method, and BAGGLO stands for biased agglomerative method; all three high-dimensional clustering methods were implemented by the CLUTO software[1, 2] (http://glaros.dtc.umn.edu/gkhome/cluto/cluto/overview).

	Our method	RB	RBR	BAGGLO
Our method	1			
RB	0.19212	1		
RBR	0.18030	0.66858	1	
BAGGLO	0.30951	0.55631	0.44082	1

Supplementary Table 8: F-measure to evaluate how similar the population substates obtained by different methods. The higher F-measure is, the more similar two methods' clustering results are. Note that RB stands for repeated bisecting K-means method, RBR stands for refined RB method, and BAGGLO stands for biased agglomerative method; all three high-dimensional clustering methods were implemented by the CLUTO software[1, 2] (http://glaros.dtc.umn.edu/gkhome/cluto/cluto/overview)

	Our method	RB	RBR	BAGGLO
Our method	1			
RB	-0.00244	1		
RBR	-0.00288	0.36789	1	
BAGGLO	-0.00894	0.17245	0.10606	1

Supplementary Table 9: Adjusted rand index (ARI) measure to evaluate how similar the population substates obtained by different methods. The higher ARI is, the more similar two methods' clustering results are. Note that RB stands for repeated bisecting K-means method, RBR stands for refined RB method, and BAGGLO stands for biased agglomerative method; all three high-dimensional clustering methods were implemented by the CLUTO software[1, 2] (http://glaros.dtc.umn.edu/gkhome/cluto/cluto/overview)

Supplementary Note 1: Computational method of identifying frequent spatial clusters

After obtaining a population of M=10,000 3D genome structures from TCC data, each with the same N=428 chromatin domains, we identify the frequent spatial clusters: sets of chromatin regions (from one or multiple chromosomes) that spatially co-localize in a reasonable percentage of the population (at least 1%). This problem has unique properties, and to our knowledge, has not been addressed before in the literature.

Problem description

Because the human genome has two sets of chromosomes, each domain has two homologous copies, which are modeled as two identical spheres in the 3D genome structures. We transform each 3D genome structure into a *chromatin interaction graph* (*CIG*) with 2N nodes, where each node represents a domain copy and each edge denotes an interaction between domain copies. As shown in Figure 1 of the main text, each node in a *CIG* has three labels: (L1) the index of the chromosome where the domain is from; (L2) the position index of the domain within its chromosome; and (L3) a label indicating which of the two chromosome copies the domain comes from (Supplementary Figure 2a). Therefore, in the triplet L1-L2-L3, $L1$ and L3 indicate whether or not two domains reside in the same homologous chromosome copy. For example, nodes 1-1-A and 1-3-B represent two different domains of chromosome 1 located in different chromosome copies (A and B). After modeling each 3D genome structure as a *CIG*, the frequent spatial cluster problem can be formed as the problem of discovering frequent dense subgraphs across many *CIGs*. However, the diploid genome brings a novel feature to the problem of identifying frequent spatial clusters: we must differentiate cases where a cluster contains two domains from the same or different chromosome copies. This novel property of *CIGs* is called "coupled isomorphism", and is different from the classic property of "graph isomorphism". Coupled isomorphism has not been addressed before in the literature. Specifically, while the fact of having "twin" chromosome copies already gives rise to "classic isomorphism", the situation where *multiple* domains can be distributed among the same or different chromosome copies constitutes "coupled isomorphism". Supplementary Figure 2 illustrates the difference between "coupled isomorphism" and "classic isomorphism". In summary, each node has two different types of labels. L1 and L2 are explicit labels indicating the genomic location (i.e., which position in which chromosome) of a domain. L3 is an implicit label indicating which chromosome copy the domain comes from. The explicit labels are shared by all graphs/structures, whereas the implicit label is "coupled-isomorphic", meaning that it must be dynamically mapped – the $L3$ labels are never shared across graphs. They can only be compared within a specific graph to decide if domains reside in the same of different chromosome copies. For example, copy A of chromosome 1 in graph 1 does not necessarily correspond to copy A of chromosome 1 in graph 2. This "coupled isomorphism", along with the large number and scale of the graphs (tens of thousands of graphs, each with hundreds of nodes), poses a great challenge.

Therefore, we developed an efficient computational framework with four steps:

Step 1: Transform each 3D genome structure into a chromatin interaction graph.

Step 2: Defer the coupled isomorphism problem by contracting the graphs to combine homologous nodes. The contracted graphs are guaranteed to preserve all occurrences of frequent patterns in the original graphs. We will revisit the coupled

isomorphism at a later step when only small patterns are processed, effectively reducing the problem complexity.

Step 3: Use our previously developed, tensor-based integrative graph mining method[3] on the contracted chromatin interaction graphs to identify frequent dense subgraphs.

Step 4: For each subgraph discovered in Step 3, replace its nodes with the original nodes and labels to recover frequent spatial clusters in the original isomorphic chromatin interaction graphs.

The pipeline of our graph mining algorithm is shown in Figure 1 of the main text. We elaborate on each step as follows.

Constructing chromatin interaction graphs from 3D genome structures (Step 1)

Step 1 is to transform each 3D genome structure into a chromatin interaction graph (*CIG*). Each homologous domain (modeled as a sphere) is represented as a node in the *CIG*. Given two spheres *i* and *j* with coordinate vectors $\vec{P}_i$, $\vec{P}_j$ and radii R_i , R_j , their relative distance is $d(\vec{P}_i,\vec{P}_j)=\frac{\|\vec{P}_i-\vec{P}_j\|}{R_i+R_j}$. If this value is less than 2, we define a chromatin interaction between the two spheres. The reason we set the relative distance threshold as 2 is because in our structural modeling procedure, if two domains have their center-to-center distance less than 2 times of the sum of their radii, the two domains were enforced to form a contact. Setting the same edge threshold allows a consistent transformation of a population of genome structures to a population of chromatin interaction networks.

Node contraction (Step2)

Step 2 is to contract homologous nodes of the *CIG* into a single node. This step reduces the number of nodes by half. As shown in Supplementary Figure 5, we contract the graph by (1) merging each pair of "twin" nodes (homologous domains) with labels *L*1-*L*2-*A* and *L*1-*L*2-*B* into one node with the label *L*1-*L*2, and (2) connecting two nodes *i* and *j* in the contracted *CIG* (*cCIG*) with an edge if there exists at least one edge in the original *CIG* between nodes *i-A* (or *i-B*) and *j-A* (or *j-B*). Koyuturk *et al.* first proposed the concept of graph contraction for mining frequent subgraphs in graphs with isomorphism [4]. Although our graphs have more complicated node labels than those used by Koyuturk *et al.*, we can guarantee that contraction preserves all frequent dense subgraphs in the *CIG* (see Theorems 1 and 2).

Theorem 1 (Preservation of dense subgraphs). *Given a chromatin interaction graph CIG and its contracted graph cCIG, any dense subgraph (in which only one of "twin nodes" can be included) in CIG is a dense subgraph in cCIG. Specifically, the density of a subgraph in cCIG is always equal to or greater than the densities of its counterparts in CIG.*

Proof. Given a subgraph (denoted as *S*) of *CIG* where only one of "twin nodes" can be included, its contracted subgraph (denoted as *cS*) in *cCIG* should have the same size (i.e., number of nodes) as *S*. According to the node contraction procedure, any edge in *S* should have a corresponding edge in *cS*. This leads to the fact that the number of edges in *cS* should be equal to or greater than the number of edges in *S*. Because the number of nodes in *S* and *cS* are the same, we have the conclusion that the density of a subgraph in *cCIG* is always equal to or greater than the densities of its counterparts in *CIG*.■

Corollary 1 (Preservation of frequent dense clusters) *Given a set of chromatin interaction graphs* $\mathbf{G}$*=*$\{CIG_1, \cdots, CIG_M\}$ *and the set of their contracted graphs contract(*$\mathbf{G}$*)=*$\{cCIG_1, \cdots, cCIG_M\}$*, any frequent dense subgraph in* $\mathbf{G}$ *is a frequent dense subgraph in contract(*$\mathbf{G}$*). Specifically, the frequency of a pattern in contract(*$\mathbf{G}$*) is always equal to or greater than the frequency of its counterpart in* $\mathbf{G}$.

The theorem and the corollary can be interpreted as follows. In a set of contracted graphs (*cCIGs*), the frequent dense clusters are a superset of the frequent dense clusters in the original set of chromatin interaction graphs (*CIGs*). Therefore, we miss no frequent dense subgraphs when mining the contracted graphs.

Since the contracted graphs don't have isomorphism, we can apply our previously developed tensor-based method[3] to efficiently identify frequent dense subgraphs, then recover frequent dense clusters in the original graphs. Using this strategy, we only need to consider the isomorphism problem for the small identified patterns.

Tensor-based frequent dense subgraph identification algorithm (Step 3)

Step 3 is to apply our tensor-based, integrative graph mining method[3] on the contracted chromatin interaction graphs to identify frequent dense subgraphs. This algorithm employs a 3rd-order tensor to model multiple graphs and iteratively optimize the objective function for pattern discovery. Since this method uses a tensor-based optimization procedure, it is very efficient, easy to use, and requires only three thresholds to determine how large, dense and frequent a target pattern should be.

A simple counting algorithm for final pattern recovery (Step 4)

Step 4 is to recover frequent dense subgraphs/clusters in the original chromatin interaction graphs from the contracted subgraphs obtained in Step 3. We use a simple counting method for this step, since most of the contracted subgraphs are small (the average size is ~5 nodes). Given a contracted subgraph with N domains and C chromosomes and occurring in K contracted graphs, we perform the following procedure (Supplementary Figure 6): **(i)** the contracted pattern is expanded to the set of 2^N possible equivalent subgraphs in the original *CIG*s, since each domain can choose one of the 2 twin nodes. In the example of Supplementary Figure 6, N=4 and C=2. Since *all occurrences of a contracted pattern share the same domain location indexes (i.e., L1-L2),* we simplify the labels at this step to use only *L3* copy indexes (A or B). That is, if two domain copies (e.g., 1-1-A and 1-2-A) of Chromosome 1 are located in the same copy A, their labels can be simplified to the sequence of copy indexes "A, A". For example, a subgraph of 4 domains is expanded to 2^4=16 subgraphs, labeled with different 4-letter sequences (e.g., "A, A, B, B"). We then categorize these 16 subgraphs into 2^{N-C}=4 isomorphic groups, each with 2^C=4 subgraphs. For example, in "isomorphic group 1", all domains of chromosome 1 are in the same copy, and all domains of chromosome 2 are also in the same copy. Likewise, in "isomorphic group 4", all domains of chromosome 1 are from different chromosome copies, and all domains of chromosome 2 are also from different chromosomal copies. **(ii)** For a given original graph (*CIG*), if an isomorphic group contains at least one subgraph whose density exceeds the chosen threshold (e.g., 0.6), we select this group as an occurrence of the contracted pattern in this original graph. Therefore, we find one or more isomorphic groups with occurrences of the pattern in each original graph. **(iii)** Based on the isomorphic groups in each original graph found in step ii, we count the number of original

graphs in which each isomorphic group occurs, and choose the most frequent isomorphic group (i.e., the one which occurs in the most original graphs) as our solution. For example, Isomorphic Group 1 occurs in 800 out of 1000 original graphs, and is the most frequent. **(iv)**We find the dense subgraphs within the most frequent isomorphic group (e.g., Group 1) and output them as the recovered dense subgraphs. This counting approach is very efficient because: (1) All patterns are very small, so they can be loaded into memory for a fast "subgraph density" computation. (2) The more chromosomes a pattern contains, the smaller the number of isomorphic groups 2^{N-C} (i.e., the less "coupled isomorphism" in the pattern), and the faster the algorithm. (3) Since this recovery process is processed on a single pattern, we can recover all the patterns in parallel. (4) In most cases, the subgraph density computation of step ii does not need to exhaustively check all possible subgraphs in the group, and it stops at the first dense subgraph.

Experimental setting

In Step 3, we identified 4452 frequent dense subgraphs in the contracted graphs, each of which contains ≥ 4 nodes, appears in ≥ 100 graphs (i.e., 1% of the 10,000 genome structures in the population) and has a density ≥ 0.8. Theorems 1 and 2 guarantee that the contracted subgraphs identified in Step 3 may have a number of occurrences which are not dense in the original graphs. Therefore, if we had used a more lenient density threshold (≥0.6) in Step 3, a much larger number of contracted patterns would have been discovered, but many of these would be false positives (i.e., their corresponding subgraphs in the original CIGs would not satisfy density threshold and consequently they would not meet the minimum frequency criterion). This is why we used a strict density criterion (≥0.8) in Step 3, but the standard density criterion for the recovered subgraphs in Step 4 (≥0.6). Using this strategy, we recovered 3856 frequent dense subgraphs in the original graphs, each of which contains at least 4 nodes, has an edge density at least 0.6, and occurs in at least 100 genome structures.

Supplementary Note 2: Effects of different combinations of parameters on final outcomes of frequent spatial clusters

To test the influence of the parameters on our result, we now use different parameter sets (minimum size, minimum density, and minimum frequency) to identify clusters. The minimum cluster size was chosen from 4 to 10, the minimum density was chosen from {0.5, 0.55, 0.6, 0.65, 0.7, 0.75, 0.8}, and the minimum frequency was chosen from {100, 200, 300, 400, 500, 600, 700, 800}. In total, we had 392 different combinations of parameter sets, and we identified frequent spatial clusters under each of them.

As expected, as the minimum size, minimum density, or minimum frequency threshold increases, the number of identified clusters decreases. Also as expected, we found that the percentage of inter-chromosomal clusters in the identified clusters decreases as the minimum frequency, the minimum density, and the minimum size threshold increases (Supplementary Figure 7). This is because on average, the frequency/density/size of intra-chromosomal clusters was much higher than that of inter-chromosomal clusters. While the number of clusters (and inter-chromosomal clusters) decrease when more stringent parameter settings were applied, we found that our major conclusions from the downstream functional analyses hold despite the diverse parameter settings. To demonstrate this, we detail our results on the following 48 parameter combinations that provide sufficient numbers of clusters for functional analysis: the minimum cluster size was chosen from {4, 5, 6}, the minimum density from {0.5, 0.55, 0.6, 0.65}, and the minimum frequency from {100, 200, 300, 400}. The number of clusters identified under these parameter settings ranges from 366 to 4716. We have the following consistent conclusions across the varied parameter settings:

(1) Many inter-chromosomal clusters are enriched with binding of one or more transcription factors.

We found that in general, as the cluster density increases, the percentage of inter-chromosomal clusters, which are enriched with at least one transcription factor, also increases (Supplementary Figure 8). Our results indicate that clusters that are highly co-localized are more likely to serve as regulatory communities. Consistent with the high percentage (58.3%) of TF-enriched inter-chromosomal clusters reported in the main manuscript, the percentage of TF enriched inter-chromosomal clusters under different parameter settings ranged from 47.4% to 82.9%, with the median percentage being 68.5%.

We also compared the top enriched transcription factors for each inter-chromosomal cluster set under different parameter combinations. In all 48 cluster sets, 10 TFs consistently occupied the top enriched list (Supplementary Table 3). Among the 10 TFs, 8 were from TF-Group 2, including c-Fos, RFX5, USF2, WHIP, NF-kB, SIN3, TBP, and IRF3; 2 were from TF-Group 3, including RNAPII and NFYB; there were no TFs from TF-Group 1 or TF-Group 4.

(2) Many inter-chromosomal clusters were enriched in centromeric interactions.

In the main manuscript, inter-chromosomal clusters with at least 30% of domains being centromeric domains were categorized as being under strong centromeric influence, and we identified a high percentage (60.5%) of inter-chromosomal clusters under strong centromeric influence. Consistently, under different parameter settings, the percentage of inter-chromosomal clusters under strong centromeric influence ranged from 42.5% to 78.5%, and the median percentage was 64.9%.

Also, under all the parameter combinations, our results consistently support that centromeric domains are hubs for inter-chromosomal associations. Firstly, the closer a domain is to the centromere of its chromosome, the more frequently it participates in inter-chromosomal clusters (Supplementary Figure 9a). Secondly, clusters involving more chromosomes generally have a higher proportion of centromeric domains, as exemplified by two parameter settings in Supplementary Figure 9b (the minimum size=4, the minimum density=0.6, and minimum frequency=300; the minimum size=5, the minimum density=0.5, and the minimum frequency=200. The number of inter-chromosomal clusters under the two parameter combinations was 1893 and 2477 respectively).

(3) The binding of TFs to chromatin clusters show functional-specific groupings (e.g. activators, repressors, and immune response TFs), and clusters enriched in different TF groups show different spatial distributions in the nucleus.

For each of the 48 parameter settings, we clustered TFs based on their enrichment profiles across the chromatin clusters, as described in the main manuscript. In almost all settings we observed three dominant TF groups, and those groups were largely overlapped with the three TF groups we reported in the main manuscript (Supplementary Figure 10a). The Jaccard Similarity Coefficient was used to measure the TF overlapping percentage. For TF-Group1, the overlapping percentage ranged from 55.6% to 100% with the median 100%. For TF-Group 2, the overlapping percentage ranged from 50% to 100% with the medium 90.9%. For TF-Group 3, the overlapping percentage ranged from 64.3% to 100% with the medium 78.6%. The low-end of the overlapping percentages were all due to the limited number of inter-chromosomal clusters identified under the extreme conditions.

For different parameter combinations, we also checked the radial positions of the inter-chromosomal clusters that were enriched with the three different groups of transcription factors, and found that they exhibit patterns similar to those reported in main text (Supplementary Figure 10b). Results indicate our conclusions about the grouping of the TFs and their nuclear location preferences are not sensitive to the parameter settings.

Supplementary Note 3: Functional annotation of frequent spatial clusters

To investigate the functional roles of the spatial clusters, we collected the genome-wide ChIP-seq data for 74 transcription factors from Encode[5] and DNA replication timing data[6] for human lymphoblastoid cells. To test whether a TF is enriched in a given cluster, we used the permutation test to generate 1000 random clusters satisfying two constraints: (1) the number of chromosomes and the number of centromeric domains in each chromosome are the same as in the given cluster; and (2) the relative domain order is the same as in the given cluster, to account for the linear domain-distance effect on spatial genome organization. For example, if the cluster we are testing contains three domains {3, 5, 8} from chromosome 1, then we generate a random cluster with three domains chosen from a random chromosome, but the randomly chosen domains must be separated by 2 and 3 domains. For example, one random instance might be domains {34, 36, 39} from chromosome 2. We generated 1000 sets of random clusters, and each set contains 3856 random clusters matching the aforementioned criteria. For each cluster we quantify the *p*-value of its enrichment in the binding of a certain TF by comparing its TF signal to those in random clusters. Then for each TF, FDR adjustment[7] was applied to all the clusters' *p*-values assessing the binding enrichment of this TF, and only clusters with *q*-value < 0.05 are reported as enriched with this TF. The number of clusters enriched with each TF was shown in Supplementary Figure 11. We found that TFs are more likely to be enriched in inter-chromosomal clusters than in intra-chromosomal clusters.

In addition, we used the DNA replication data from Repli-seq at cell cycle stages G1 and G2 to annotate early and late DNA replication clusters. Firstly, we calculated DNA replication signals at G1 stage for all the domains using the UCSC utility bigWigSummary, where the signal was normalized by domain size. Then we calculated DNA replication signal at G1 stage for a given cluster by taking the average signal of all the domains in the cluster. After obtaining DNA replication signal at G1 stage for all the clusters, we performed the same permutation test followed by FDR adjustment (q-value < 0.05) to define early DNA replication clusters. Similarly, we defined late DNA replication clusters by using DNA replication signal at G2 stage.

Supplementary Note 4: Comparison with the clusters directly identified from the Hi-C contact map

To compare our frequent spatial clusters with the clusters directly identified from the Hi-C contact map (hereafter referred as map-based clusters), we employed two popular overlapping graph clustering algorithms (linkcomm[8] and commDetNMF[9]) with various algorithm parameters that work on a single graph. We took the following steps for the comparison:

Step 1. Generate binary contact graph: firstly, we scale the contact frequency g_{ij} between two domains *i* and *j* to the value a_{ij} in the range between 0 and 1, which reflect the probability of their contact. The scale function was used in [10]and defined as below:

$$a_{ij} = \begin{cases} \dfrac{g_{ij}}{\min(f_i^{\max}, f_j^{\max})}, & if \ \dfrac{g_{ij}}{\min(f_i^{\max}, f_j^{\max})} \leq 1 \\ 1, & otherwise \end{cases}$$

where $f_i^{\max} = \min(g_{i(i-1)}, g_{i(i+1)})$ indicating the minimum contact frequency of domain *i* to its left and right neighbor domains (the domains *i-1* and *i+1*) in genomic sequence (we assume that the two neighbor domains shall always be in contact). Secondly, given the scaled contact map $A = (a_{ij})_{428\times428}$, we used different edge cutoffs, $e = 0.1, 0.2, 0{,}3, 0.4, 0.5, 0.6, 0.7, 0.8, or\ 0.9$, to convert *A* to a binary contact map $C = (c_{ij})_{428\times428}$, such that $c_{ij} = 1$ when $a_{ij} \geq e$, otherwise $c_{ij} = 0$. For example, $C^{(0.3)}$ denotes a binary contact map that records all contacts whose scaled contact frequency ≥ 0.3. Each binary contact map $C^{(e)}$ is actually a graph, in which clusters can be identified by an overlapping graph clustering algorithms. So we regard the edge cutoff *e* as a parameter of the graph clustering algorithm.

Step 2. Identify clusters in each binary contact map using different overlapping graph clustering algorithms and parameters: two popular overlapping graph clustering algorithms were employed with their various algorithm parameters, i.e., linkcomm[8] and commDetNMF[9]. linkcomm has the parameter “partition density threshold” that is used for cutting the dendrogram to obtain clusters[8]. We performed linkcomm by varying this algorithm parameter as 0.1, 0.2, 0.3, 0.4, 0.5, 0.6, 0.7, 0.8, and 0.9. We removed duplicates from all clusters identified with these parameters. We impose two basic criteria on the clusters: (1) “minimum cluster size”, i.e. *n*, requires that the clusters must have at least *n* domains in a cluster; we varied *n* as 4, 5, 6, 7, 8, 9, 10, 11, 12, 13, 14, 15; and (2) “minimum edge density of subgraph”, i.e., *d*, requires a cluster’s edge density must be $\geq d$, and we varied it as 0.5, 0.6, 0.7, 0.8, and 0.9. For example, the cluster set with *algorithm*=linkcomm, *e*=0.3, *d*=0.7 and *n*=6, includes all clusters identified by linkcomm on the binary contact graph $C^{(0.3)}$, each of which has at least 6 domains and edge density≥0.7. Therefore, linkcomm can identify $9 \times 5 \times 12 = 540$ cluster sets (some may be empty), each of which is from a parameter combination (*e*, *d*, *n*).

commDetNMF is a popular non-negative matrix factorization based overlapping graph clustering method, by which each factorization component (a

vector) represents a cluster (i.e., the larger value an element of this vector is, the more likelihood its corresponding domain belongs to the cluster). As the outcome of this algorithm is dependent on the number of components and the initialization (due to its coordinate descend optimization scheme)[9], we performed commDetNMF with 10 times of random initialization for each predefined number of components (i.e., 50, 100, 200 400, 600, 800, 1000, 1200, 1400, 1600). We then use these factorization components to obtain the clusters that have at least *n* domains and with the edge density at least *d*. We varied *n* as 4, 5, 6, 7, 8, 9, 10, 11, 12, 13, 14, 15 and varied *d* as 0.5, 0.6, 0.7, 0.8, 0.9. Therefore, commDetNMF can identify $9 \times 5 \times 12 = 540$ cluster sets (some may be empty), each of which results from a parameter combination (*e*, *d*, *n*).

Step 3. Overlap comparison between the Hi-C contact map derived clusters and our 3856 frequent spatial clusters. We conduct overlap comparison between all map-derived clusters by a clustering algorithm and our frequent spatial clusters. We denote our 3856 frequent spatial clusters as the cluster set $A = \{a_1, \ldots, a_{3856}\}$ and denotes as the cluster set $B = \{b_1, b_2, \ldots\}$ all the map-derived clusters that are identified by a particular algorithm under different parameter settings. The overlap between two clusters (denoted as *a* and *b*) is evaluated by a popular measure of set similarity, i.e., Jaccard Index, defined as $J(a, b) = \frac{|a \cap b|}{|a \cup b|}$. So two clusters are regarded as overlap with each other if $J(a, b) \geq \theta$. We could vary overlap threshold θ=0.6, 0.9 for loosed or stringent cluster overlap definitions. Therefore, given a cluster overlap criterion ($J(a, b) \geq \theta$), in this step, we perform the overlap comparison between *A* and *B* by calculating the fraction of frequent spatial clusters in *A* that have overlap with Hi-C contact map derived clusters in *B*, i.e., $\frac{|A \cap B|}{|A|}$.

Step 4. Compare TF binding enrichment. We performed the TF binding enrichment for the map-derived cluster set *B* obtained in Step 3. The TF binding enrichment analysis followed the same procedure as described in Supplementary Section 3. We examined the TF binding enrichment rates of three cluster subsets: the clusters shared by the map-based clusters and our frequent spatial clusters (denoted as the set "$A \cap B$"), the clusters that are uniquely identified by our structure-based method (denoted as the set "unique *A*"), and the clusters that are uniquely identified by the map-based clustering method (denoted as the set "unique *B*").

Supplementary Table 4 and Supplementary Table 5 list the comparison results based on two cluster overlap definitions: $\theta = 0.6, 0.9$ for the overlap criterion $J(a, b) \geq \theta$, respectively. According to these comparison results, we have the following conclusions:

(1) **Most of the frequent spatial clusters cannot be identified by the map-based clustering algorithms.** Based on the loose cluster overlap criterion ($J(a, b) \geq 0.6$), as shown in Supplementary Table 4 , clusters identified by the algorithm "linkcomm" with different parameters account for only 12.5% of our frequent spatial clusters; and those by the algorithm "commDetNMF" with different parameters account for only 14.1% of our

frequent spatial clusters. Moreover, as shown in Supplementary Table 5, using the stringent cluster overlap criterion (for $J(a, b) \geq 0.9$), the map derived clusters can only account for less than 1% of our frequent spatial clusters.

(2) **The clusters uniquely identified by our method (the set "unique *A*") have much higher TF enrichment rates than the clusters uniquely identified from the Hi-C contact map (the set "unique *B*").** Based on the loose cluster overlap criterion ($J(a, b) \geq 0.6$), as shown in Supplementary Table 4 , the frequent spatial clusters uniquely identified by our method have ~55% TF enrichment rate; for the algorithm "linkcomm" ("commDetNMF"), 30.4% (33.0%) of the identified clusters are enriched with the binding of at least one TF, and among its all unique clusters (that are not FSCs under the loose cluster overlap criterion), 33.1% (35.2%) of which are enriched with the binding of at least one TF.

Supplementary Note 5: Testing the significance of centromere-centromere colocalization

We applied the colocalization test [11]on all triplet combinations of different centromeres, to examine whether the majority of triplet-centromeres showed significant *p*-values of colocalization based on the Hi-C data.

Paulsen et al.[11] considered four different structural features to preserve the interaction dependency: sequence-based distances, transitivity relations, domain structures, and regional preferences. Randomly chosen regions should have the same structure features as the target regions. Since our target set are subcentromeric regions from different chromosomes, we didn't consider the first three structure features, because sequence-based distances and transitivity relations only apply to intra-chromosomal interactions. The reason we didn't consider domain structure features is because what we are testing is whether subcentromeric regions have higher co-localization than other regions on the same chromosome. If we test whether certain subcentromeric regions have higher co-localization than other subcentreomeric regions, domain structure feature should be considered. For the last structure feature regional preferences, Paulsen et al. refers to that two genomic elements in the same relative position on the chromosome are more likely to interact than genomic elements on different positions. We followed exact the same procedure in the paper to partition the chromosome arms into six equally sized groups, and generated random regions that have the same relative positions on the chromosomes.

We used the iterative Hi-C correction method [12] to obtain the normalized Hi-C contact matrix at 100 kb resolution, and then used this matrix to test whether sub-centromeric regions have significant co-localization, compared to other regions on the same chromosomes.

We tested each triplet-chromosome set for significant co-localization on sub-centromeric regions. In total, the number of triplet-chromosome set from 23 chromosomes is 1771. For each set, we calculated the average of normalized contact frequency on sub-centromeric regions, then normalized it by adjusting for expectation and standard deviation (for inter-chromosomal interactions, the expectation and standard deviation are constant). To access the significance of sub-centromeric co-localization signal, we randomly retrieved genomic regions of the same length that are not positioned at sub-centromeres on the same chromosome, and calculated the co-localization signal. We repeated the procedure 1000 times and calculated the permutation *p*-values, which were subsequently adjusted by FDR for the multiple testing correction.

We found that the majority of triplet-chromosome showed significant colocalization in sub-centromeric regions from the Hi-C data (Supplementary Table 6).

Supplementary Note 6: Measuring the partial correlation between TF enrichment and cluster frequency

Centromere-centromere clustering plays an important role for inter-chromosomal organization, and inter-chromosomal clusters with strong centromeric influence had higher frequency than inter-chromosomal clusters with weak centromeric influence (first boxplot of Figure 4e). In order to examine the effect of transcription factors binding in cluster stability, the influence of centromeres on cluster frequency should be removed. We computed the partial correlation between cluster frequency and the number of significantly enriched TFs in inter-chromosomal clusters, after accounting for the proportion of centromeric domains in the cluster.

We denote the number of enriched TFs in each inter-chromosomal cluster by X, the frequency of each the inter-chromosomal clusters by Y, and the proportion of centromeric domains in each inter-chromosomal cluster by Z. The partial correlation $\gamma_{XY \bullet Z}$ was calculated as

$$r_{XY \cdot Z} = \frac{r_{XY} - r_{XZ} r_{YZ}}{\sqrt{1 - r_{XZ}^2}\sqrt{1 - r_{YZ}^2}}$$

Supplementary Note 7: Measuring the correlation between TF signal and centromere contact frequency

Each chromosome may contain multiple centromeric domains, which are defined as domains that overlap with the subcentromeric region (5Mb up- and downstream to centromere locations). We measured the TF signal on the subcentromeric region of a chromosome as the averaged signal across all the centromeric domains on that chromosome. After we obtained TF signal on subcentromeric regions for all the chromosomes, we performed the quantile-quantile normalization[13], because the 11 TFs in Group 2 had different signal distributions. After the quantile-quantile normalization, we took the average of TF signal across all the 11 TFs for each subcentromeric region, which represented the average TF signal in Group 2 on the subcentromeric regions (x-axis in Figure 5f).

We used the domain-domain contact matrix (dimensions 856×856) from our 3D genome structures to calculate the subcentromere-subcentromere contact frequencies. The entry of the matrix ranged from 0 to 10000, where 0 indicates that two corresponding domains do not form a contact in any structure, and 10000 indicates the two domains form a contact in all the 10000 structures. For a given chromosome, we first obtained all the centromeric domains (defined as domains that overlap subcentromeric regions), and then calculated the average contact frequency of each centromeric domain with all the other centromeric domains from different chromosomes. Among all the contact frequencies of centromeric domains (of a chromosome) with the other centromeres, we retrieved the maximum as the respective sub-centromere's contact frequency with all the other sub-centromeres (y-axis in Figure 5f).

Supplementary Note 8: Clusters-based and contacts-based partition generate different sub-populations of genome structures

We constructed a contact-based vector to describe each genome structure: the vector has $\binom{428}{2} = 91378$ elements, each of which represents a domain pair with a binary value 0 or 1 to indicate if two domains contact. Then we used 10,000 such high-dimensional binary vectors to group genome structures into 8 population substates. Note that the text document data is similar to this data, because they also have high-dimensionality and can be represented by binary values. The problem of how to effectively cluster extremely high-dimensional text data has been extensively studied in the text mining field. Therefore, instead of using naïve k-means clustering, we used a popular high-dimensional data clustering software – CLUTO[1] from that field.

Specifically, we used three efficient clustering methods in the CLUTO software with default parameters and the number of clusters K=8: repeated bisecting K-means (abbreviated as RB), refined RB (abbreviated as RBR), and biased agglomerative (abbreviated as BAGGLO) methods. The first two use partition clustering strategy and the last uses agglomerative clustering strategy. They all have been evaluated in the top-ranking machine learning and data mining journals[1, 2] and were recommended for high-dimensional data clustering task.

We used three popular clustering validation evaluation measures, which were designed by three different principles: (1) information theory based measure – normalized mutual information (NMI), (2) set overlap based measure – F-measure, and (3) counting pairs based measure – adjusted rand index (ARI). All three measures are symmetric, indicating they have commutative property, so we report only one value when comparing two methods. Results are shown in Supplementary Tables 7-9.

All of the above comparisons arrive at the same conclusion: using frequent chromatin clusters as features can yield quite different clustering results from those using all possible contacts as features.

[1] CLUTO software: http://glaros.dtc.umn.edu/gkhome/cluto/cluto/overview

Supplementary References

1. Zhao, Y. & Karypis, G. Empirical and theoretical comparisons of selected criterion functions for document clustering. *Mach Learn* **55**, 311-331 (2004).
2. Zhao, Y. & Karypis, G. Hierarchical clustering algorithms for document datasets. *Data Mining and Knowledge Discovery* **10**, 141-168 (2005).
3. Li, W. et al. Integrative analysis of many weighted co-expression networks using tensor computation. *PLoS Comput Biol* **7**, e1001106 (2011).
4. Koyuturk, M., Kim, Y., Subramaniam, S., Szpankowski, W. & Grama, A. Detecting conserved interaction patterns in biological networks. *Journal of computational biology : a journal of computational molecular cell biology* **13**, 1299-1322 (2006).
5. Consortium, E.P. et al. An integrated encyclopedia of DNA elements in the human genome. *Nature* **489**, 57-74 (2012).
6. Hansen, R.S. et al. Sequencing newly replicated DNA reveals widespread plasticity in human replication timing. *Proceedings of the National Academy of Sciences of the United States of America* **107**, 139-144 (2010).
7. Strimmer, K. fdrtool: a versatile R package for estimating local and tail area-based false discovery rates. *Bioinformatics* **24**, 1461-1462 (2008).
8. Ahn, Y.Y., Bagrow, J.P. & Lehmann, S. Link communities reveal multiscale complexity in networks. *Nature* **466**, 761-764 (2010).
9. Psorakis, I., Roberts, S., Ebden, M. & Sheldon, B. Overlapping community detection using Bayesian non-negative matrix factorization. *Physical review. E, Statistical, nonlinear, and soft matter physics* **83**, 066114 (2011).
10. Kalhor, R., Tjong, H., Jayathilaka, N., Alber, F. & Chen, L. Genome architectures revealed by tethered chromosome conformation capture and population-based modeling. *Nat Biotechnol* **30**, 90-98 (2012).
11. Paulsen, J. et al. Handling realistic assumptions in hypothesis testing of 3D co-localization of genomic elements. *Nucleic acids research* **41**, 5164-5174 (2013).
12. Imakaev, M. et al. Iterative correction of Hi-C data reveals hallmarks of chromosome organization. *Nature methods* **9**, 999-1003 (2012).
13. Bolstad, B.M., Irizarry, R.A., Astrand, M. & Speed, T.P. A comparison of normalization methods for high density oligonucleotide array data based on variance and bias. *Bioinformatics* **19**, 185-193 (2003).
